# Strategic interaction of original equipment manufacturers between outsourcing and purchasing in a quality-differentiated market

**DOI:** 10.1371/journal.pone.0262678

**Published:** 2022-01-18

**Authors:** Dan Yang, Sen Liu, Zhe Zhang, Fu Huang, Jingyang Wang

**Affiliations:** 1 International Business School, Yunnan University of Finance and Economics, Kunming, China; 2 School of Logistics, Yunnan University of Finance and Economics, Kunming, China; 3 School of Economics and Management, Nanjing University of Science & Technology, Nanjing, China; 4 School of Economics and Management, Huizhou University, Huizhou, China; Univerza v Mariboru, SLOVENIA

## Abstract

Economic globalization has swept the whole world. To focus on their main business, enterprises that are referred to as original equipment manufacturers (OEMs) outsource non-core production activities to contract manufacturers (CMs). By constructing a two-level supply chain consisting of two competing OEMs and one upstream CM, the strategic interaction of the OEMs between outsourcing and purchasing is studied. Specifically, the CM can offer custom- and predefined modes of original equipment manufacturing (namely, CO mode and PO mode, respectively). The former mode enables OEMs to determine product quality, while the latter only allows them to purchase from several quality configurations. The results show that, first, since the CO mode allows the adopter to lead the product design, whether to choose this mode depends on the required R&D cost. Interestingly, however, a lower R&D cost does not necessarily result in the adoption of the CO mode if the product quality difference is small under the PO mode. Second, the optimal purchasing strategy of an OEM is indifferent to the outsourcing mode (CO and PO) of its rival but significantly affected by the quality cost. However, compared to the PO mode, choosing the CO mode would cause the competitor to suffer more profit losses. Third, differing from the prior literature, this paper finds that when the downstream OEM can make quality decisions, although this may lead to profit loss of the contract manufacturer in some channels, it could benefit the CM overall.

## 1. Introduction

In the context of globalization, the world economy has become integrated; any enterprise only exists as a certain link or several links in the supply chain, and some low-value businesses are outsourced to other organizations to complete. For example, to minimize supply risks and reduce production costs, Apple outsources 70% of its iPhone production business to Foxconn and the remaining 30% to Pegatron Group [1]. In the PC industry, the notebook computers of Apple, Dell, Sony and Toshiba are mainly manufactured by Taiwan’s Asus [2]. In 2010 alone, Asian firms created over $110 billion of output value in OEM business [3]. As a result, outsourcing activities have become an important part of a company’s operations.

However, in April 2018, the US government announced that ZTE was forbidden to purchase products involving sensitive technologies from American enterprises. Since the core system and key components are highly dependent on foreign suppliers, this "blocking" action seriously damaged the production and operation activities of ZTE, especially the mobile phone business, which was nearly shut down [4]. In cell phone industries, technology giants, such as Xiaomi, Vivo, Oppo and ZTE, as original equipment manufacturers (OEMs), generally take charge of product concepts, shapes and structure design, while other processes, such as component procurement, product assembly and testing, are outsourced to contract manufacturers (CMs), such as Foxconn and BYD [5]. The "ZTE incident" reminded OEMs to rethink the rationality of this outsourcing mode. For the sake of comparison, we call it the predefined OEM mode in this paper.

Another mode is the so-called custom OEM. Similar to the predefined OEM, most of the component procurement and product assembly are also entrusted to a CM; the difference is that in addition to the overall design of the product, the OEMs are responsible for the R&D of the key component (e.g., the central processing unit of a smartphone). Under the custom OEM mode, the core technology or component of the product is the focus of the enterprise, and other non-core businesses can be outsourced. Taking the mobile phone industry as an example, Huawei chose to design the core SoC (system-on-chip) of mobile phones by itself and outsourced its chip production to TSMC, the world’s leading semiconductor manufacturer. Benefitting from this strategy, Huawei can focus on the demands of customers and integrate them into SoC development. The Kirin 970 processor developed by Huawei is the world’s first mobile phone chip with a built-in independent neural network unit, which can greatly improve the performance of mobile phone photography and thus benefit customers who value photography functions [6]. However, competitors such as Xiaomi and Oppo are limited by their SoC suppliers, and they can only catch up to Huawei when Qualcomm provides an upgraded Snapdragon chip [7]. Obviously, strong control over key components of production makes the custom OEM model more competitive.

There is no doubt that taking control of product design can enhance the competitiveness of firms [8, 9]. However, choosing the custom OEM mode means investing in the R&D of the core technology, and whether this is a worthwhile effort is a hard question to answer. First, this investment requires sufficient funds. In 2019, Huawei’s operating revenue exceeded 800 billion yuan, and its R&D investment surpassed 100 billion yuan, most of which was related to the mobile phone processor developed in-house. Meanwhile, the revenue of Xiaomi and OPPO in the same period was only 1/4 of that of Huawei [10]. Second, even if companies can perform their R&D in-house, they need an incentive to make that decision. For instance, Nokia’s mobile phone market share was over 25% in 2013, ranking first in the world, but it still chose to purchase chips from Texas Instruments instead of custom OEM [11]. Some studies have suggested that, in a market with homogenized products, enterprises’ enthusiasm for R&D investment will be inhibited [12].

To address this research gap, the strategic interaction of original equipment manufacturers between outsourcing and purchasing should be further studied. This study mainly discusses the following questions: 1) What are the conditions for a manufacturer choosing the custom OEM mode? Is the custom mode always optimal when the required R&D investment is low? 2) How does a manufacturer make purchase decisions in a quality-differentiated supplying market? How does the competitor’s OEM mode affect its choice? 3) How do downstream manufacturers’ OEM mode and procurement strategy affect the upstream CM?

The rest of this paper is organized as follows. Section 2 reviews the related literature. Section 3 describes the problem and basic model setting. Section 4 analyses four models and shows the optimal order quantity and profits of the supply chain members under the four models. In Section 5, we discuss the optimal strategy choice of *M*_1_ and *M*_2_ and the impact of strategy choice on upstream CM. Finally, conclusions and managerial insights are given in Section 6.

## 2. Literature review

### 2.1 Outsourcing decision-making

The first stream of literature related to our paper is the original equipment manufacturer’s outsourcing decision-making. Some scholars focus on the influential factors of outsourcing. Wang et al. [13] investigated how the pricing sequence affects the competition between contract manufacturers and original equipment manufacturers in the end market and found that when the wholesale price is exogenous, either party prefers to be a Stackelberg leader. Zhu [14] designed an optimal outsourcing contract involving product cost, quality and time to market and considered both complete and incomplete information, which aims to model the buyer’s internal variable cost information. Leoni [15] pointed out that with the continuous increase in investment, if the marginal probability of successful product research and development converges smoothly to zero, then even if the compensation for R&D failure is certain and there exists an optimal contract equilibrium, outsourcing R&D work still has only a small chance of innovation success. Feng et al. [16] studied how the relationship between the cost of providing fast service and the cost of achieving high-quality service affects outsourcing contracts and supply chain performance. The results show that when the cost is positively correlated, simple contracts usually perform well, but when the cost is negatively correlated, contracts with complex structures need to be designed. The other part of the study is mainly about the choice of outsourcing strategy. Shen et al. [17] compared the application of OEM and ODM (original design manufacturer) outsourcing modes in the fashion industry and found that when suppliers trade with retailers through wholesale price contracts under the ODM strategy, suppliers have no incentive to invest in innovation. Bolandifar et al. [18] and Xu et al. [19] both studied the impact of OEMs outsourcing procurement to contract manufacturers on supply chain members. The former work suggested that contract manufacturers could still benefit even if they did not lead the procurement, while the latter found that the outsourcing strategy of multinational companies was affected by local competitors and international tax rules. Chen et al. [20] studied the outsourcing relationship between OEMs and CMs in the same product market and explored the win-win conditions under which OEMs and CMs support outsourcing in all cases.

As shown in Table 1, the differences between the prior studies and our paper are summarized in two aspects: 1) the literature has only studied the predefined OEM mode, whose access to manufacturer’s leading core technology research and development of the custom OEM mode is not allowed. While the custom OEM mode has become an important means of outsourcing high-tech enterprises, a full comparison of the two kinds of OEM modes is discussed in this paper, specifically, pricing competition, order of decision-making, contract manufacturers and the impact of supply chain member benefits. 2) Outsourcing and procurement are important production activities of manufacturing enterprises. This study first investigates the strategic interaction of original equipment manufacturers between OEM mode selection and procurement strategy selection.

**Table 1:** Summary of the related literature regarding outsourcing decision-making.

Literature	Original Equipment Manufacturing Mode		Competition of OEMs
	Predefined	Custom	
Wang et al. [13]	✓		
Zhu [14]	✓		
Leoni [15]	✓		
Feng et al. [16]	✓		
Shen et al. [17]	✓		
Bolandifar et al. [18]	✓		
Xu et al. [19]	✓		
Chen et al. [20]	✓		
Our study	✓	✓	✓

### 2.2 Product quality differentiation in competition

The second literature stream involves the topic of product quality differentiation in competition. Jaskold and Thisse [21] and Mussa and Rosen [22] have made pioneering contributions to address the impact of vertical differentiation on manufacturers’ product selection and pricing under a fixed quality level. Shaked and Sutton [23], from the perspective of supply chain coordination, found that differentiated product strategies could help alleviate supply chain conflicts. In recent years, Chen et al. [24] studied how manufacturers with flexible production capacity determine the best product quality difference, price and scheduling when consumers can evaluate product quality. Li and Chen [25] found that exogenous quality differences intensify retailer price competition in the Stackelberg game, while endogenous quality differences could improve the performance of the whole supply chain. In terms of information asymmetry, Li et al. [26] proposed an adaptive spatial network model for manufacturers’ pricing decisions in the face of uncertain product quality and customer learning. Keskin and Birge [27] discussed how companies construct product lines considering the uncertain cost of quality. In addition, some studies have focused on the interaction between quality differentiation and firms’ nonprice operating decisions. Huang et al. [28] showed that, in a duopoly, if market followers have more cost advantages than leaders in terms of quality improvement, they can benefit from strategic decentralization. Li and Chen [29] studied the backward integration strategy between retailers and different manufacturers, believing that retailers are more willing to integrate with upstream suppliers with high exogenous quality; otherwise, retailers should integrate with companies with low quality. In addition, Cui [30] and Jain and Bala [10] studied the impact of investment strategy on differentiated competition. The former group indicated that reasonable quality improvement of leaders could weaken the motivation of followers to free ride, while the latter found that providing differentiated services does not always alleviate homogeneous competition for quality-differentiated products.

As shown in Table 2, differing from the above studies, this paper combines quality differentiation with enterprise procurement and outsourcing strategy, discusses what quality configuration to purchase when competitors choose different OEM modes and analyses the impacts of these strategic interactions on supply chain performance.

**Table 2:** Summary of the related literature regarding product quality differentiation in competition.

Literature	Supply Chain Operational Topics						
	Pricing	Quality Control	Coordination	Channel/Power Structure	Investment Strategy	Procurement	Outsourcing
Jaskold and Thisse [21]	✓						
Mussa and Rosen [22]	✓						
Shaked and Sutton [23]	✓		✓				
Chen et al. [24]	✓	✓					
Li and Chen [25]	✓	✓					
Li et al. [26]	✓						
Keskin and Birge [27]	✓			✓			
Huang et al. [28]				✓			
Li and Chen [29]		✓		✓			
Cui [30]		✓			✓		
Jain and Bala [12]					✓		
Our Study	✓	✓				✓	✓

## 3. Problem description

As a world-famous semiconductor manufacturer, Samsung not only sold Exynos chips designed and manufactured by itself for downstream mobile phone OEM firms (such as Vivo), but also provided chip customization services (such as the A-Series SoC of Apple iPhone) [31]. Motivated by this, consider a two-echelon supply chain consisting of one upstream contract manufacturer (CM) and two downstream original equipment manufacturers, where the CM, such as Samsung, can provide two OEM modes: (1) Predefined OEM mode (PO strategy), CM undertakes all activities of product production from design to assembly, and it produces a two-type product for the strategy adopter to choose, i.e., a high-quality item (*i* = *H*) with quality level *q*_*H*_ = 1 and a low-quality item (*i* = *L*) with quality level *q*_*L*_ < 1; (2) Custom OEM mode (CO strategy), the adopter would lead R&D of the core component and thus could determine the product quality level *q* (*q*_*L*_ < *q* < *q*_*H*_ = 1). As a result, the CM is only responsible for implementing the adopter’s manufacturing plan. Note that theoretically, CM can certainly add countless quality configurations, but it is obviously impractical to consider the unlimited possibilities. What we want to emphasize is that the quality configuration (*q*_*L*_ and *q*_*H*_) of CM is predetermined, and all firms understand this knowledge. The adopter who chooses custom mode will determine the quality level under their own profit maximization.

For clarity, the parameters and variables involved in this paper are summarized in Table 3.

**Table 3:** Parameters and variables and their definitions.

Parameters	Definition
j	Both *j* and *M*_*j*_, *j* ∈ {1, 2}, denote the manufacturer entities downstream of CM
$\overset{-}{j}$	The set of all the members in {1, 2} that do not belong to *j*, *j* ∈ {1, 2}
*i*	The product type offered by contract manufacturer, *i* ∈ {*L*, *H*}, and *i* = *H*(*L*) denotes the high (low) quality item.
*P*_*j*_	Product price of manufacturer *M*_*j*_, *j* ∈ {1, 2}
$P_{\overset{-}{j}}$	Product price of manufacturer $M_{\overset{-}{j}}$, $\overset{-}{j}\in \left\{1,2\right\}$
*q*_*H*_	Predefined high-quality level, 0 < *q*_*L*_ < *q*_*H*_ = 1
*q*_*L*_	Predefined low-quality level
*q*_*j*_	Product quality level of manufacturer *M*_*j*_, 0 < *q*_*j*_ < 1
*a*	Basic product demand, *a* > 1
*α*	Substitution coefficient of *M*_1_ and *M*_2_, 0 < *α* < 1
*m*	The amortized R&D cost of contract manufacturer, *m* > 0
*c*	*M*_1_’s quality cost coefficient, *c* > 0
*f*	Raw material cost of contract manufacturer, *f* ≤ *c*
*k*	The fixed R&D expenditure of *M*_1_, *k* > 0
Variables	Definition
*Q*_*j*_	Order quantity of manufacturer *M*_*j*_, *Q*_*j*_ > 0
*w*_*j*_	Wholesale price offered to manufacturer *M*_*j*_, *w*_*j*_ > 0
π_j_	Profit of manufacturer *j*, π_j_ > 0
*q*	The quality level decided by M1 when chooses HO strategy, *q*_*L*_ < *q* < *q*_*H*_

**Fig 1:**
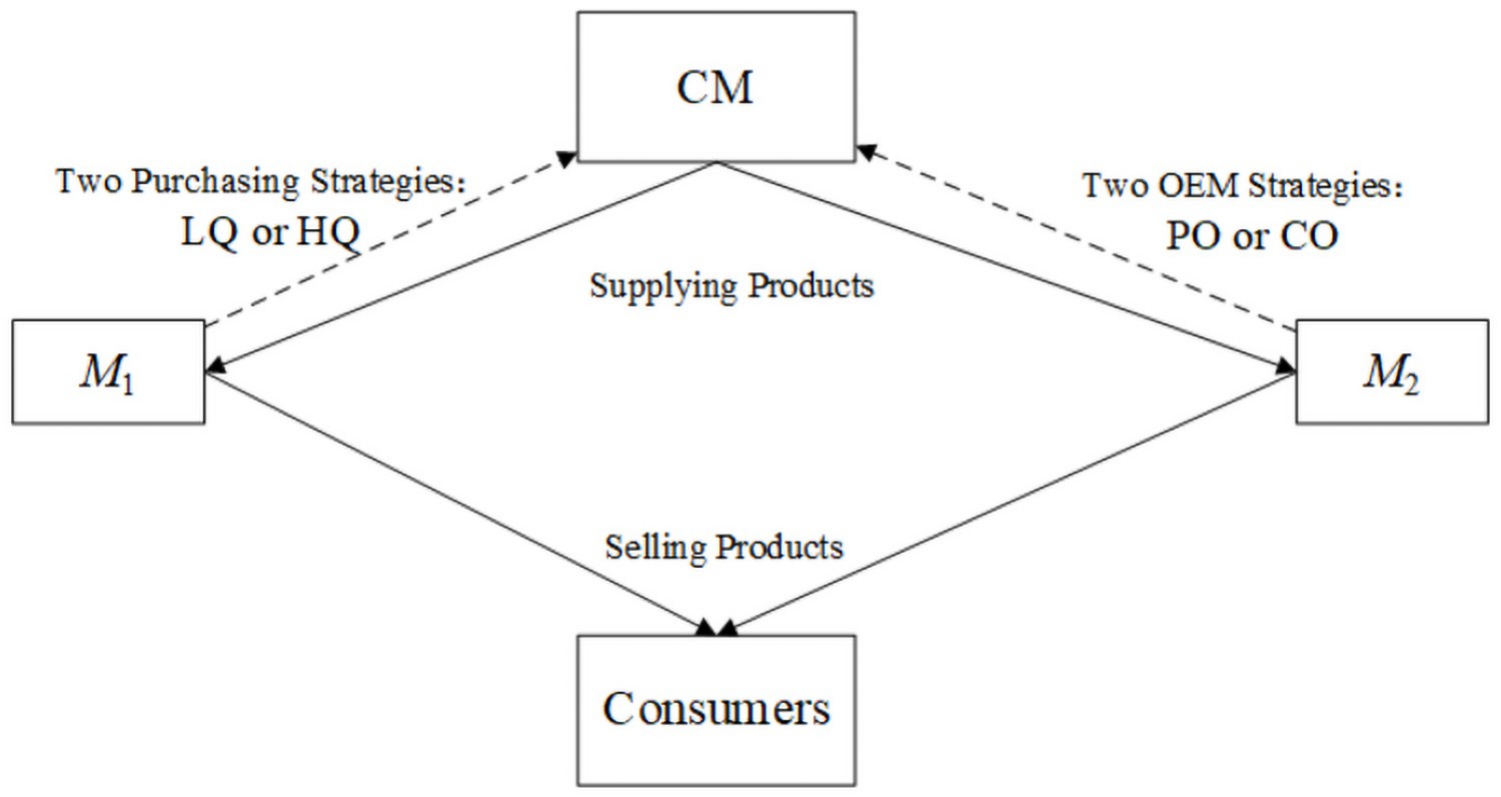
The relationship between the contract manufacturer and the original equipment manufacturers.

We assume that the market power of the two manufacturers is different. In particular, the dominant manufacturer (*M*_1_, such as Huawei whose products are equipped with Kirin chips developed by itself and SoC purchased from Media Tek) can choose different OEM modes (PO or CO strategy). As a market leader, if choosing the PO mode, *M*_1_ will only consider purchasing high-quality products (*q*_1_ = *q*_*H*_ = 1). In contrast, the disadvantaged manufacturer (*M*_2_, such as Vivo) cannot provide financial support for R&D and hence only chooses the PO mode that needs to decide the procurement strategy: buying high-quality products (HQ strategy, *q*_2_ = *q*_*H*_ = 1) or buying low-quality products (LQ strategy, *q*_2_ = *q*_*L*_ < 1). Furthermore, we visualize the relationship between the contract manufacturer and the two original equipment manufacturers in Fig 1.

Since there are only *M*_1_ and *M*_2_ suppliers in the end market, we employ a linear demand function that is affected by the price and quality of its own product and the price and quality of competitive products, referring to classical duopoly firm theory [31–37], the product demand function can be expressed as $Q_{j}=a-P_{j}+\alpha P_{\overset{-}{j}}+q_{j}-\alpha q_{\overset{-}{j}}$. Note that the units of all variables and parameters are not considered in this function, since such a stylized model (please see https://en.wikipedia.org/wiki/Stylized_fact, some scholars may call it to abstract model) is not data-driven, and its purpose is to reveal the impact of price and other factors on demand through a simple but quantitative method. Similar dimensionless functions were commonly used in the literature regarding management science. For an instance, Banker et al. [37] employed *q*_*i*_ = *k*_*i*_*α* − *βp*_*i*_ + *γp*_*j*_ + *λx*_*i*_ − *μx*_*j*_ to draw the linear impact of product *i*’s price *p*_*i*_ and quality *x*_*i*_ and product *j*’s price *p*_*j*_ and quality *x*_*j*_ on the former’s demand *q*_*i*_. In addition, we thank anonymous reviewers for their comments, and remind readers who want to use real data to drive the stylized models that we assume all quantities in equations *Q*1 and *Q*2 to be dimensionless or having normalized units. It is common not to specify units of measurement in such models, the right member of the demand equality contains a coefficient for each term, so there is the expected conversion from the number of products to prices and quality level.

In the demand function of $Q_{j}=a-P_{j}+\alpha P_{\overset{-}{j}}+q_{j}-\alpha q_{\overset{-}{j}}$, $\overset{-}{j}$ ($\overset{-}{j}\in \{1,\;2\}$) is the set of all the members in {1, 2} that do not belong to *j* (*j* ∈ {1, 2}), if *j* = 1, $\overset{-}{j}=2$ otherwise *j* = 2, $\overset{-}{j}=1$; *a* refers to the potential market demand, which also reflects the total market size, and it is much larger than other parameter to avoid cases with negative demand [33, 38]; *Q*_*j*_ refers to the order quantity of *M*_*j*_, which reflects the consumer demand for its products; *q*_*j*_ refers to the quality level of *M*_*j*_ products; *P*_*j*_ ($P_{\overset{-}{j}}$) represents the market price of the *M*_*j*_ *($M_{\overset{-}{j}}$*) product; *α* (0 < *α* < 1) is the substitution coefficient or so-called cross elasticity coefficient in classical microeconomic literature [39], which characterizes the impacts of rival product’s price and quality on its own demand, and reflects the competition degree between the two products [34, 38]. Thus, we have the related product demands in the different channels:

$$Q_{1}=a-P_{1}+\alpha P_{2}+q_{1}-\alpha q_{2},Q_{2}=a-P_{2}+\alpha P_{1}+q_{2}-\alpha q_{1}$$ (1)

**Fig 2:**
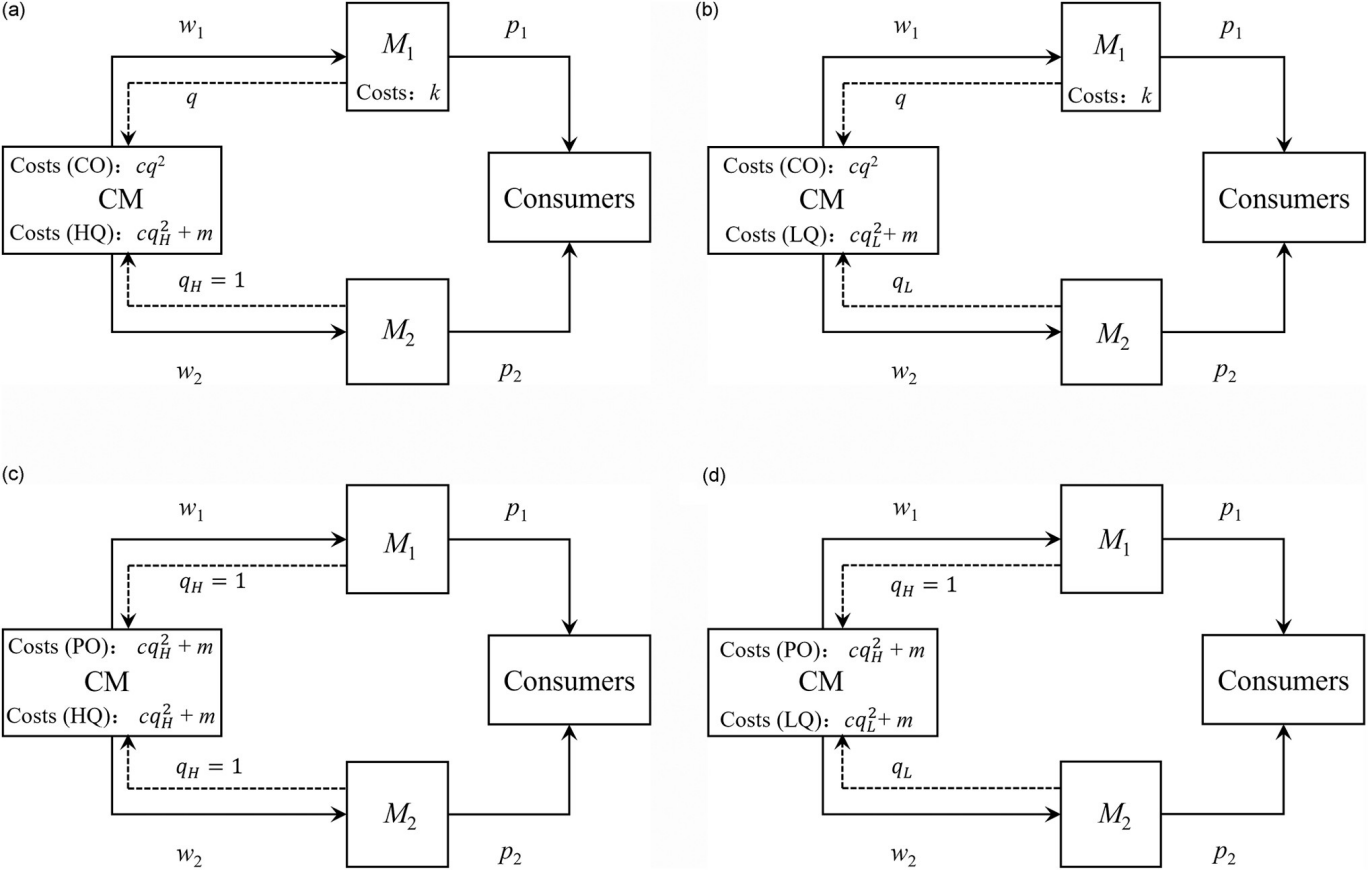
Four strategy combinations. (a) COHQ model, (b) COLQ model, (c) POHQ model, and (d) POLQ model.

In PO mode, the unit production cost of the CM is composed of raw material cost *f* and amortized R&D cost *m* (i.e., R&D cost per unit product). Following the literature [34, 40–42], we adopt a quadratic function of raw material cost to characterize the serious impact of improving unit product quality on cost input: $f=cq_{i}^{2}$, where *c* is the quality cost coefficient, which reflects the CM’s production technology. *M*_1_ and *M*_2_ purchase from the CM at *w*_1_ and *w*_2_ unit wholesale prices, respectively. In CO mode, the unit cost of the CM only includes *f*. Note that the adopter of CO mode leads the R&D of the core component, so it has the related invention patents. As a result, it does not need to pay *m* for CM. Since *M*_1_ is responsible for the development of core technologies, it also needs to pay fixed expenditures *k* for the equipment, personnel and places needed for the research and development. Finally, the four strategy combinations with detailed model settings are shown in Fig 2.

## 4. Model analysis

### 4.1 *M*_1_ chooses the CO mode, and *M*_2_ sells high-quality products (*q*_1_ = *q*, *q*_2_ = 1)

In the COHQ model, *M*_1_ leads the design and development of its own products, while the CM only provides OEM production services for it. Due to its weak strength, *M*_2_ can only choose the PO mode and compete with *M*_1_ by purchasing high-quality products from the CM. Following the literature [14, 43], the decision order is as follows: First, *M*_1_ determines the quality level of its products *q*; second, the CM determines the wholesale prices *w*_1_ and *w*_2_ according to the quality requirements of *M*_1_ and its own manufacturing capacity. Finally, *M*_1_ and *M*_2_ determine the order quantities *Q*_1_ and *Q*_2_, respectively. By solving simultaneous Eq (1), we can obtain the inverse demand functions *P*_1_(*Q*_1_, *Q*_2_) = [*a*(1 + *α*) + *q*_1_(1 − *α*^2^) − *Q*_1_ − *Q*_2_*α*]/(1 − *α*^2^) and *P*_2_(*Q*_1_, *Q*_2_) = [*q*_2_(1 − *α*^2^) + *a*(1 + *α*) − *Q*_2_ − *Q*_1_*α*]/(1 − *α*^2^). According to Section 3, *q*_1_ = *q* and *q*_2_ = 1 in this case. Based on the backward induction method [34, 41, 44], we first solve the optimal order quantity decisions of the two OEMs, and the profit maximization problem of *M*_1_, *M*_2_ and CM can be expressed as:

$$\pi_{1}(Q_{1},Q_{2})=\left(P_{1}(Q_{1},Q_{2})-w_{1}\right)\cdot Q_{1}-k=\left[\frac{Q_{1}+Q_{2}\alpha -a\left(1+\alpha \right)+q\left(\alpha^{2}-1\right)}{\alpha^{2}-1}-w_{1}\right]Q_{1}-k\to \underset{Q_{1}}{Max}$$ (2)

$$\pi_{2}(Q_{1},Q_{2})=\left(P_{2}(Q_{1},Q_{2})-w_{2}\right)\cdot Q_{2}=\left[\frac{Q_{2}-a\left(1+\alpha \right)+\alpha \left(Q_{1}+\alpha \right)-1}{\alpha^{2}-1}-w_{2}\right]Q_{2}\to \underset{Q_{2}}{Max}$$ (3)

$$\pi_{\text{CM}}(w_{1},\;w_{2})=\left(w_{1}-cq^{2}\right)*Q_{1}(w_{1},w_{2})+(w_{2}-c-m)*Q_{2}(w_{1},w_{2})\to \underset{w_{1},w_{2}}{Max}$$ (4)

It is easy to prove that *π*_1_(*Q*_1_) and *π*_2_(*Q*_2_) are concave functions with respect to *Q*_1_ and *Q*_2_ (*π*_1_ is quadratic in *Q*_1_ and *π*_2_ is quadratic in *Q*_2_), respectively. By the first derivative condition ($\frac{\partial \pi_{1}}{\partial Q_{1}}=0$, $\frac{\partial \pi_{2}}{\partial Q_{2}}=0$), the optimal order quantity decisions are derived under the profit maximization of *M*_1_ and *M*_2_ (Note that the main derivations of this paper are derived by computer programs. We provide the copy of the source code with 4.1 Section in the Supporting Information to show how to solve optimal decisions, and the other cases can also be solved by the similar method. The readers could run it through Mathematics with 11.0 or higher version):

$$Q_{1}(w_{1},w_{2})=\frac{(1+\alpha )\left[a\left(\alpha -2\right)+(\alpha -1)(2q-2w_{1}+\left(w_{2}-1\right)\alpha )\right]}{\alpha^{2}-4}$$ (5)

$$Q_{2}(w_{1},w_{2})=\frac{(1+\alpha )\left[a\left(\alpha -2\right)+(\alpha -1)(2-2w_{2}-q\alpha +w_{1}\alpha )\right]}{\alpha^{2}-4}$$ (6)

Eqs (5) and (6) are substituted into CM’s profit maximization problem. We provide proof to illustrate that *π*_*CM*_(*w*_1_, *w*_2_) is a jointly concave function with respect to *w*_1_ and *w*_2_ (Appendix 9 in S1 Appendix). The optimal wholesale price decision of CM can be obtained as follows:

$$w_{1}(q)=\frac{1}{2}\left(q+cq^{2}+\frac{a}{1-\alpha }\right)$$ (7)

$$w_{2}=\frac{1}{2}\left(1+c+m+\frac{a}{1-\alpha }\right)$$ (8)

Similarly, it is easy to prove that *π*_1_(*q*) is a concave function with respect to *q*. Substituting Eqs (5)–(8) into Eq (2), the optimal quality decision can be obtained by solving *M*_1_’s profit maximization problem $\pi_{1}(q)=\left(P_{1}(q)-w_{1}(q)\right)*Q_{1}(q)-k\to \underset{q}{Max}$. By solving $\frac{\partial \pi_{1}\left(q\right)}{\partial q}=0$ can find there exists three roots. However, the second root is always negative, and the third root exceeds 1 under constrains that *a* (the basic market demand) is much larger than other parameter to avoid cases with negative demand [33, 38], then we have:

$$q=\frac{1}{2c}$$ (9)

According to Eqs (2)–(9), we have Theorem 1:

**Theorem 1**: In the COHQ model, the optimal decisions and benefits of *M*_1_, *M*_2_ and CM are shown in Appendix 1.1 in S1 Appendix.

### 4.2 *M*_1_ chooses the CO mode, and *M*_2_ sells low-quality products (*q*_1_ = *q*, *q*_2_ = *q*_*L*_)

In the COLQ model, *M*_1_ still dominates the design and development of its products; *M*_2_ chooses to purchase the CM’s low-quality products to compete with *M*_1_. The decision order is the same as the COHQ model. First, *M*_1_ determines the quality level of its products *q*. Second, the CM determines the wholesale prices *w*_1_ and *w*_2_ according to the quality requirements of *M*_1_ and its own manufacturing capacity. Finally, *M*_1_ and *M*_2_ determine their respective order quantities *Q*_1_ and *Q*_2_, respectively. Referring to Eq (1), we can obtain the inverse demand functions *P*_1_(*Q*_1_, *Q*_2_) = [*a*(1 + *α*) + *q*_1_(1 − *α*^2^) − *Q*_1_ − *Q*_2_*α*]/(1 − *α*^2^) and *P*_2_(*Q*_1_, *Q*_2_) = [*q*_2_(1 − *α*^2^) + *a*(1 + *α*) − *Q*_2_ − *Q*_1_*α*]/(1 − *α*^2^). According to Section 3, *q*_1_ = *q* and *q*_2_ = *q*_*L*_ in this case. Based on the backward induction method, we first solve the optimal order quantity decisions of the two OEMs, and the profit maximization problem of *M*_1_ and *M*_2_ can be expressed as:

$$\pi_{1}(Q_{1},Q_{2})=\left(P_{1}(Q_{1},Q_{2})-w_{1}\right)\cdot Q_{1}-k=\left[\frac{Q_{1}+Q_{2}\alpha -a\left(1+\alpha \right)+q\left(\alpha^{2}-1\right)}{\alpha^{2}-1}-w_{1}\right]Q_{1}-k\to \underset{Q_{1}}{Max}$$ (10)

$$\pi_{2}(Q_{1},Q_{2})=\left(P_{2}(Q_{1},Q_{2})-w_{2}\right)\cdot Q_{2}=\left[\frac{Q_{2}+Q_{2}\alpha -a\left(1+\alpha \right)+q_{L}\left(\alpha^{2}-1\right)}{\alpha^{2}-1}-w_{2}\right]Q_{2}\to \underset{Q_{2}}{Max}$$ (11)

$$\pi_{CM}\left(w_{1},\;w_{2}\right)=\left(w_{1}-cq^{2}\right)*Q_{1}\left(w_{1},w_{2}\right)+\left(w_{2}-cq_{L}^{2}-m\right)*Q_{2}(w_{1},w_{2})\to \underset{w_{1},w_{2}}{Max}$$ (12)

It is easy to prove that *π*_1_(*Q*_1_) and *π*_2_(*Q*_2_) are concave functions with respect to *Q*_1_ and *Q*_2_, respectively. By the first derivative condition ($\frac{\partial \pi_{1}}{\partial Q_{1}}=0$, $\frac{\partial \pi_{2}}{\partial Q_{2}}=0$), the optimal order quantity decisions are derived under the profit maximization of *M*_1_ and *M*_2_:

$$Q_{1}(w_{1},w_{2})=\frac{(1+\alpha )\left[a\left(\alpha -2\right)+(\alpha -1)(2q-2w_{1}+(w_{2}-q_{L})\alpha \right]}{\alpha^{2}-4}$$ (13)

$$Q_{2}(w_{1},w_{2})=\frac{(1+\alpha )\left[a\left(\alpha -2\right)+(\alpha -1)(2q_{L}-2w_{2}+(w_{1}-q)\alpha \right]}{\alpha^{2}-4}$$ (14)

Eqs (13) and (14) are substituted into the CM’s profit maximization problem. Similar to Section 4.1, it is easy to prove that *π*_*CM*_(*w*_1_, *w*_2_) is a jointly concave function with respect to *w*_1_ and *w*_2_, and the optimal wholesale price decision of the CM can be obtained as follows:

$$w_{1}(q)=\frac{1}{2}\left(q+cq^{2}+\frac{a}{1-\alpha }\right)$$ (15)

$$w_{2}=\frac{1}{2}\left(m+q_{L}+cq_{L}^{2}+\frac{a}{1-\alpha }\right)$$ (16)

Similarly, it is easy to prove that *π*_1_(*q*) is a concave function with respect to *q*. Substituting Eqs (13)–(16) into Eq (10), the optimal quality decision can be obtained by solving *M*_1_’s profit maximization problem $\pi_{1}=\left(P_{1}(q)-w_{1}(q)\right)*Q_{1}(q)-k\to \underset{q}{Max}$, and by the same solution process as the model of COHQ we have:

$$q=\frac{1}{2c}$$ (17)

According to Eqs (10)–(17), we have Theorem 2:

**Theorem 2**: In the COLQ model, the optimal decisions and benefits of *M*_1_, *M*_2_ and CM are shown in Appendix 1.2 in S1 Appendix.

### 4.3 *M*_1_ chooses PO mode, and *M*_2_ sells high-quality products (*q*_1_ = 1, *q*_2_ = 1)

In the POHQ model, *M*_1_ gives up the core technology research and development of investment products and, like M2, chooses to purchase high-quality products from a CM for OEM sales. In this case, the decision order of all parties is simpler than the previous two modes. First, the CM determines the wholesale prices *w*_1_ and *w*_2_. Next, *M*_1_ and *M*_2_ determine the order quantities *Q*_1_ and *Q*_2_, respectively. Referring to Eq (1), we can obtain the inverse demand functions *P*_1_(*Q*_1_, *Q*_2_) = [*a*(1 + *α*) + *q*_1_(1 − *α*^2^) − *Q*_1_ − *Q*_2_*α*]/(1 − *α*^2^) and *P*_2_(*Q*_1_, *Q*_2_) = [*q*_2_(1 − *α*^2^) + *a*(1 + *α*) − *Q*_2_ − *Q*_1_*α*]/(1 − *α*^2^). According to Section 3, *q*_1_ = *q*_2_ = 1 in this case. Based on the backward induction method, we first solve the optimal order quantity decisions of the two OEMs, and the profit maximization problem of *M*_1_ and *M*_2_ can be expressed as:

$$\pi_{1}\left(Q_{1},Q_{2}\right)=\left(P_{1}(Q_{1},Q_{2})-w_{1}\right)\cdot Q_{1}=\left[\frac{Q_{1}-a\left(1+\alpha \right)+\alpha \left(Q_{2}+\alpha \right)-1}{\alpha^{2}-1}-w_{1}\right]Q_{1}\to \underset{Q_{1}}{Max}$$ (18)

$$\pi_{2}\left(Q_{1},Q_{2}\right)=\left(P_{2}(Q_{1},Q_{2})-w_{2}\right)\cdot Q_{2}=\left[\frac{Q_{2}-a\left(1+\alpha \right)+\alpha \left(Q_{1}+\alpha \right)-1}{\alpha^{2}-1}-w_{2}\right]Q_{2}\to \underset{Q_{2}}{Max}$$ (19)

$$\pi_{CM}\left(w_{1},\;w_{2}\right)=\left(w_{1}-c-m\right)*Q_{1}\left(w_{1},\;w_{2}\right)+\left(w_{2}-c-m\right)*Q_{2}(w_{1},\;w_{2})\to \underset{w_{1},w_{2}}{Max}$$ (20)

It is easy to prove that *π*_1_(*Q*_1_) and *π*_2_(*Q*_2_) are concave functions with respect to *Q*_1_ and *Q*_2_, respectively. By the first derivative condition ($\frac{\partial \pi_{1}}{\partial Q_{1}}=0$, $\frac{\partial \pi_{2}}{\partial Q_{2}}=0$), the optimal order quantity decisions are derived under the profit maximization of *M*_1_ and *M*_2_:

$$Q_{1}(w_{1},\;w_{2})=\frac{(1+\alpha )\left[a\left(\alpha -2\right)+(\alpha -1)(2-2w_{1}+\left(w_{2}-1\right)\alpha ))\right]}{\alpha^{2}-4}$$ (21)

$$Q_{2}(w_{1},\;w_{2})=\frac{(1+\alpha )\left[a\left(\alpha -2\right)+(\alpha -1)(2-2w_{2}+\left(w_{1}-1\right)\alpha ))\right]}{\alpha^{2}-4}$$ (22)

Eqs (21) and (22) are substituted into the CM’s profit maximization problem. Similar to Section 4.1, it is easy to prove that *π*_*CM*_(*w*_1_, *w*_2_) is a jointly concave function with respect to *w*_1_ and *w*_2_, and the optimal wholesale price decision of the CM can be obtained as follows:

$$w_{1}=w_{2}=\frac{1}{2}(1+c+m+\frac{a}{1-\alpha })$$ (23)

According to Eqs (18)–(23), we have Theorem 3:

**Theorem 3**: In the POHQ model, the optimal decisions and benefits of *M*_1_, *M*_2_ and CM are shown in Appendix 1.3 in S1 Appendix.

### 4.4 *M*_1_ chooses PO mode, and *M*_2_ sells low-quality products (*q*_1_ = 1, *q*_2_ = *q*_*L*_)

In the POLQ model, *M*_1_ still does not choose independent research and development and continues to purchase high-quality products from a CM. The difference is that *M*_2_ chooses to purchase low-quality products to compete with *M*_1_. The decision order is the same as the POHQ model: first, CM determines the wholesale prices *w*_1_ and *w*_2_; then, *M*_1_ and *M*_2_ determine the order quantities *Q*_1_ and *Q*_2_, respectively. Referring to Eq (1), we can obtain the inverse demand functions *P*_1_(*Q*_1_, *Q*_2_) = [*a*(1 + *α*) + *q*_1_(1 − *α*^2^) − *Q*_1_ − *Q*_2_*α*]/(1 − *α*^2^) and *P*_2_(*Q*_1_, *Q*_2_) = [*q*_2_(1 − *α*^2^) + *a*(1 + *α*) − *Q*_2_ − *Q*_1_*α*]/(1 − *α*^2^). According to Section 3, *q*_1_ = 1 and *q*_2_ = *q*_*L*_ in this case. Based on the backward induction method, we first solve the optimal order quantity decisions of the two OEMs, and the profit maximization problem of *M*_1_ and *M*_2_ can be expressed as:

$$\pi_{1}\left(Q_{1},Q_{2}\right)=\left(P_{1}(Q_{1},Q_{2})-w_{1}\right)\cdot Q_{1}=\left[\frac{Q_{1}-a\left(1+\alpha \right)+\alpha \left(Q_{2}+\alpha \right)-1}{\alpha^{2}-1}-w_{1}\right]Q_{1}\to \underset{Q_{1}}{Max}$$ (24)

$$\pi_{2}\left(Q_{1},Q_{2}\right)=\left(P_{2}(Q_{1},Q_{2})-w_{2}\right)\cdot Q_{2}=\left[\frac{Q_{2}+\alpha Q_{1}-a\left(1+\alpha \right)+q_{L}(\alpha^{2}-1)}{\alpha^{2}-1}-w_{2}\right]Q_{2}\to \underset{Q_{2}}{Max}$$ (25)

$$\pi_{CM}\left(w_{1},\;w_{2}\right)=\left(w_{1}-c-m\right)*Q_{1}\left(w_{1},\;w_{2}\right)+\left(w_{2}-cq_{L}^{2}-m\right)*Q_{2}(w_{1},\;w_{2})\to \underset{w_{1},w_{2}}{Max}$$ (26)

It is easy to prove that *π*_1_(*Q*_1_) and *π*_2_(*Q*_2_) are concave functions with respect to *Q*_1_ and *Q*_2_, respectively. By the first derivative condition ($\frac{\partial \pi_{1}}{\partial Q_{1}}=0$, $\frac{\partial \pi_{2}}{\partial Q_{2}}=0$), the optimal order quantity decisions are derived under the profit maximization of *M*_1_ and *M*_2_:

$$Q_{1}(w_{1},\;w_{2})=\frac{(1+\alpha )\left[a\left(\alpha -2\right)+(1-\alpha )(2w_{1}-2+q_{L}\alpha -w_{2}\alpha )\right]}{\alpha^{2}-4}$$ (27)

$$Q_{2}(w_{1},\;w_{2})=\frac{(1+\alpha )\left[a\left(\alpha -2\right)+(\alpha -1)(2q_{L}-2w_{2}+w_{1}\alpha -\alpha )\right]}{\alpha^{2}-4}$$ (28)

Eqs (27) and (28) are substituted into the CM’s profit maximization problem. Similar to Section 4.1, it is easy to prove that *π*_*CM*_(*w*_1_, *w*_2_) is a jointly concave function with respect to *w*_1_ and *w*_2_, and the optimal wholesale price decision of the CM can be obtained as follows:

$$w_{1}=\frac{1}{2}\left(1+c+m+\frac{a}{1-\alpha }\right)$$ (29)

$$w_{2}=\frac{1}{2}\left(m+q_{L}+cq_{L}^{2}+\frac{a}{1-\alpha }\right)$$ (30)

According to Eqs (24)–(30), we have Theorem 4:

**Theorem 4**: In the POLQ model, the optimal decisions and benefits of *M*_1_, *M*_2_ and CM are shown in Appendix 1.4 in S1 Appendix.

### 4.5 Comparison of optimal decisions

By comparing the four models’ optimal decisions, such as the optimal order quantity, the optimal wholesale price and the optimal quality level, the following conclusions can be drawn.

**Proposition 1**: Regardless of what quality product (LQ or HQ) *M*_2_ sells, the optimal quality decision of *M*_1_ in CO mode is consistent: *q*^*COHQ*^ = *q*^*COLQ*^.

Proposition 1 shows that the optimal quality decision of *M*_1_ in CO mode is not affected by the quality of competing products. Since *M*_1_ entrusts a CM to produce as an OEM, although *M*_1_ can lead its own product design in CO mode, the quality of its products is inevitably restricted by the OEM’s technical level. Specifically, the upper limit of its quality is high-quality products in the CM, and the lower limit is low-quality products. When the quality cost is very high ($\frac{1}{2c}\leq q_{L}$), *M*_1_ chooses to produce products with the same quality as the CM’s low-quality products; in contrast, when the quality cost is very low ($\frac{1}{2c}\geq 1$), products with the same quality as the CM’s high-quality products will be produced. In addition, the existing literature shows that product homogenization will intensify price competition among products, causing losses to all manufacturers. *M*_1_ has more advantages in producing products with different qualities. Therefore, when the quality cost is moderate ($q_{L}<\frac{1}{2c}<1$), *M*_1_ chooses to produce either high-quality or low-quality CM products. At this point, regardless of the quality of products that *M*_2_ sells, *M*_1_ can differentiate itself from *M*_2_ to maximize revenue.

**Proposition 2**: *M*_1_ can obtain a higher demand for products produced in the CO mode than in the PO mode: $Q_{1}^{COHQ}>Q_{1}^{POHQ},\;Q_{1}^{COLQ}>Q_{1}^{POLQ}$.

Proposition 2 shows that *M*_1_ can increase the order quantity in CO mode compared with PO mode. In PO mode, product quality is determined by the CM, and *M*_1_ can only select a certain fixed quality type (high quality or low value) and is unable to adapt to market changes. In the CO mode, *M*_1_ has a dominant role in product design, so in the endogenous quality situation, the optimal quality level can be determined according to consumer preferences to obtain higher demand than that in the PO mode. In the mobile phone industry, manufacturers with SoC research and development capabilities are more often preferred by consumers. For example, Huawei has greatly improved the camera performance of its Mate10 phone by adding an AI computing chip to its self-developed Kirin 970SoC, which has attracted the attention of many photography enthusiasts.

**Proposition 3**: When $c<\frac{1}{1+q_{L}}$, *M*_2_ sells high-quality products with higher demand ($Q_{2}^{COHQ}>Q_{2}^{COLQ}$, $Q_{2}^{POHQ}>Q_{2}^{POLQ}$); otherwise, when $c>\frac{1}{1+q_{L}}$, selling lower-quality products is more in demand ($Q_{2}^{COHQ}<Q_{2}^{COLQ}$, $Q_{2}^{POHQ}<Q_{2}^{POLQ}$).

Proposition 3 mainly reflects the impact of the quality cost on the demand of the contracted product. Generally, the larger *c* is, the more CMs need to pay for quality inputs, and the increased cost will be transferred to *M*_2_ through increased wholesale prices. Similarly, *M*_2_ will also increase the end price of the product to compensate for the loss caused by the rising purchase cost. Especially for high-quality products, the double marginal effect will amplify the impact of the upstream quality cost on the downstream retail price. Therefore, when the quality cost is high ($c>\frac{1}{1+q_{L}}$), the demand for low-quality products sold by *M*_2_ is higher. This is because the double marginal effect will cause the high-quality products to be priced too high, thus weakening the attraction to consumers. In contrast, when the quality cost is low ($c<\frac{1}{1+q_{L}}$), the price will not be too high even if high-quality products are sold, which will be more attractive to consumers who value product quality and are sensitive to price. Therefore, the demand for high-quality products is higher than that for low-quality products.

**Proposition 4**: Regardless of *M*_1_ or *M*_2_, the wholesale price provided by CMs is not affected by the strategic choice of competitors: $w_{1}^{POHQ}=w_{1}^{POLQ}>w_{1}^{COHQ}=w_{1}^{COLQ}$, $w_{2}^{COHQ}=w_{2}^{POHQ}>w_{2}^{COLQ}=w_{2}^{POLQ}$.

Proposition 4 shows that it is not the choice of strategy but the quality of OEM products that affects the wholesale prices of *M*_1_ and *M*_2_. Specifically, regardless of whether *M*_2_ sells high-quality or low-quality products, the wholesale price of *M*_1_ will always be higher in PO mode than in CO mode. There are two main reasons. First, when *M*_1_ dominates product design, its optimal quality level decision is usually lower than that of the CM high-quality products (unless $\frac{1}{2c}\geq 1$). When c is constant, the raw material cost of products in CO mode is lower than that in PO mode. Second, if *M*_1_ chooses the PO mode, in addition to the necessary raw material cost, CMs also have to pay the R&D cost m for each unit’s product. As a result, wholesale prices are also higher in PO mode. Similarly, the wholesale price provided by CMs to *M*_2_ is also affected by product quality. Obviously, the wholesale price of high-quality goods is higher than the wholesale price of low-quality goods, so $w_{2}^{COHQ}=w_{2}^{POHQ}>w_{2}^{COLQ}=w_{2}^{POLQ}$.

## 5. Strategy discussion

This section will analyse the optimal strategy choice of *M*_1_ and *M*_2_ and the impact of strategy choice on upstream CM benefits by comparing the benefits of the two manufacturers (*M*_1_, *M*_2_) and the contract manufacturer (CM) under different modes.

### 5.1 OEM mode selection for *M*_1_

**Proposition 5**:

1. If *M*_2_ purchases high-quality products for sale, then the optimal OEM mode of *M*_1_ will be selected as follows (see appendix for *k*_1_ − *k*_6_):
  - If $\frac{1}{2c}\leq q_{L}$, when *k* < *k*_1_, the CO mode is optimal ($\pi_{1}^{COHQ}>\pi_{1}^{POHQ}$); otherwise, when *k* < *k*_1_, the PO mode is optimal when ($\pi_{1}^{COHQ}<\pi_{1}^{POHQ}$);
  - If $q_{L}<\frac{1}{2c}<1$, when *k* < *k*_2_, the CO mode is optimal ($\pi_{1}^{COHQ}>\pi_{1}^{POHQ}$); otherwise, when *k* > *k*_2_, the PO mode is optimal when ($\pi_{1}^{COHQ}<\pi_{1}^{POHQ}$);
  - If $\frac{1}{2c}\geq 1$, when *k* < *k*_3_, the CO mode is optimal ($\pi_{1}^{COHQ}>\pi_{1}^{POHQ}$); otherwise, when *k* > *k*_3_, the PO mode is optimal when ($\pi_{1}^{COHQ}<\pi_{1}^{POHQ}$).
2. If *M*_2_ purchases low-quality products for sale, the optimal OEM mode of *M*_1_ will be selected as follows (see appendix for *k*_4_, *k*_5_ and *k*_6_):
  - If $\frac{1}{2c}\leq q_{L}$, when *k* < *k*_4_, the CO mode is optimal ($\pi_{1}^{COLQ}>\pi_{1}^{POLQ}$); otherwise, when *k* > *k*_4_, the PO mode is optimal when ($\pi_{1}^{COLQ}<\pi_{1}^{POLQ}$);
  - If $q_{L}<\frac{1}{2c}<1$, when *k* < *k*_5_, the CO mode is optimal ($\pi_{1}^{COLQ}>\pi_{1}^{POLQ}$); otherwise, when *k* > *k*_5_, the PO mode is optimal when ($\pi_{1}^{COLQ}<\pi_{1}^{POLQ}$);
  - If $\frac{1}{2c}\geq 1$, when *k* < *k*_6_, the CO mode is optimal ($\pi_{1}^{COLQ}>\pi_{1}^{POLQ}$); otherwise, when *k* > *k*_6_, the PO mode is optimal when ($\pi_{1}^{COLQ}<\pi_{1}^{POLQ}$).

Proposition 5 shows how *M*_1_’s R&D expense *k* affects its choice of the optimal OEM mode in the case that *M*_2_ chooses the HQ/LQ strategy. It is easy to understand that when the required R&D investment exceeds a certain threshold (*k*_1_ − *k*_6_), the CO mode will impose significant cost pressure on *M*_1_, and such a result is unbearable for the average SME. In fact, tens of billions of dollars are needed to develop SoCs in mobile phone manufacturing, which is one of the reasons why there are only a handful of handset manufacturers in the world that have their own chips. In contrast, when the required R&D investment is lower than the threshold value, the cost pressure of *M*_1_ decreases, and he has sufficient incentive to choose the CO mode. One example is that before Google released Android to the public, few manufacturers could develop their own mobile operating systems independently. However, since Google introduced its open-source platform, Xiaomi, Meizu, Huawei, and other companies have launched their own improved versions of Android systems. The main reason is that the open source of Android has greatly reduced the cost of mobile phone system development.

**Corollary 1**: A small difference between the CM products’ quality (*q*_*L*_ and *q*_*H*_) might motivate *M*_1_ to choose the CO mode, even if the R&D investment of *M*_1_ is not high.

**Fig 3:**
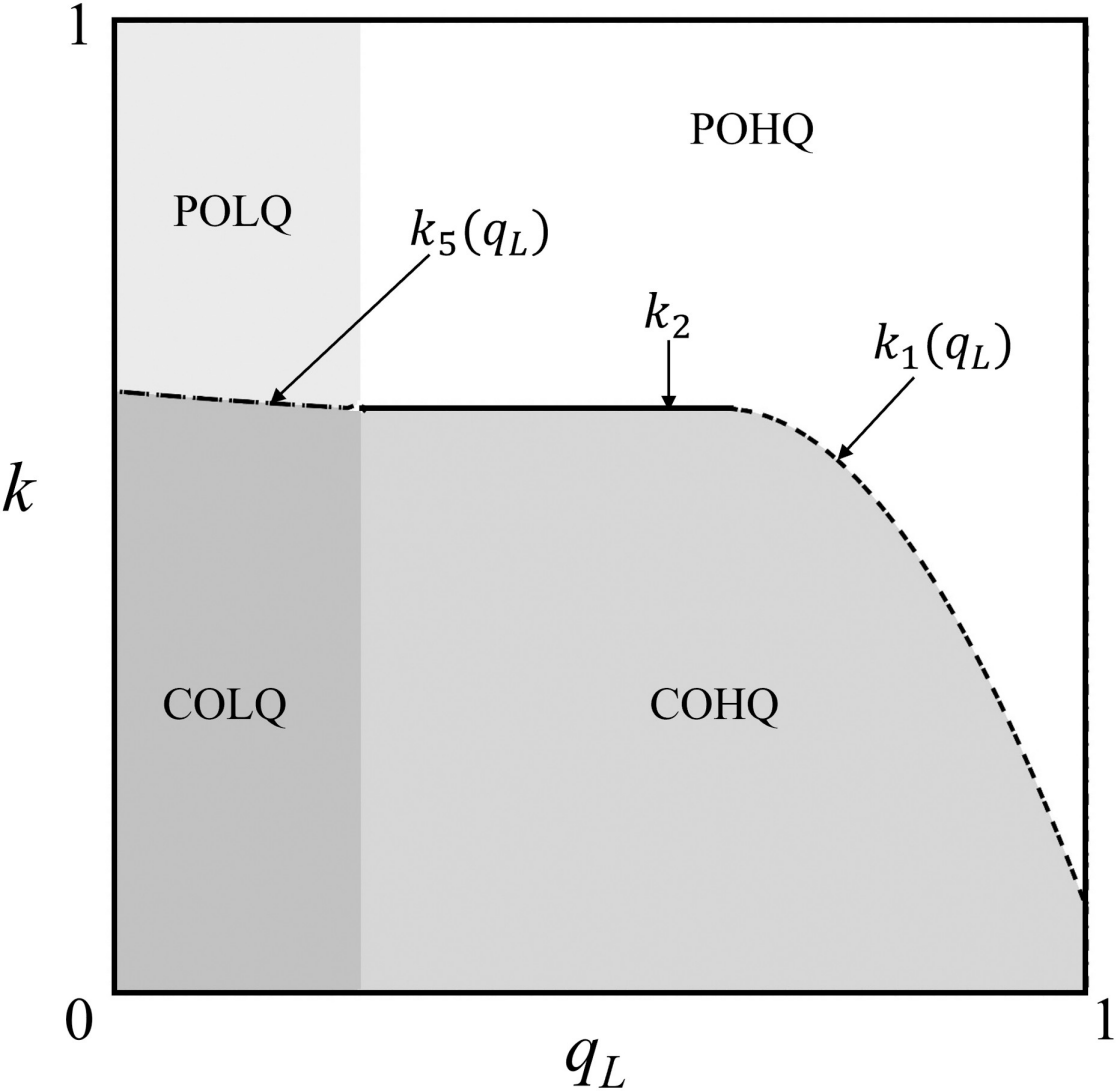
The influence of *k* and *q*_*L*_ on the maximal profit scenario of *M*_1_ (*a* = 1; *α* = 0.5; *m* = 0.02; *c* = 0.8).

Corollary 1 indicates that the quality of products provided by upstream contract manufacturers also affects the OEM model choice of downstream OEMs. As shown in Fig 3, with the increase of *k* and *q*_*L*_, maximum profit scenario of *M*_1_ might change. Note that maximum profit scenario of *M*_1_ means *M*_1_ obtain maximum profit in the scenario than in other scenarios. For example, the region of “POLQ” in Fig 3 represents $\pi_{1}^{POLQ}>Max[\pi_{1}^{COLQ},\pi_{1}^{COHQ},\pi_{1}^{POHQ}]$. The same is true in other cases.

When *M*_2_ purchases low-quality products for sale, the regions COLQ and POLQ are separated by *k*_5_ (see Proposition 5(2).b). When *M*_2_ purchases high-quality goods for sale, the boundary between the region COHQ and POHQ is a piecewise function (see Proposition 5(1)). If $\frac{1}{2c}\leq q_{L}$, the boundary *k*_1_(*q*_*L*_) is a decreasing curve with respect to *q*_*L*_. Since the quality of CM high-quality products is fixed at 1, a larger *q*_*L*_ means a smaller quality difference between high-quality products and low-quality products. (1) When *k* is small, with the decrease in the CM product quality difference, the maximum profit scenario of *M*_1_ will change from COHQ to POHQ, and its optimal strategy will change from CO to PO. The reason is that the decrease in *q*_*L*_ will lead to decreased quality choices for *M*_1_; that is, CMs would be unable to produce finished products that meet the quality requirements of *M*_1_ due to their limited technical expertise. Therefore, even though the required R&D cost is low, the optimal choice for *M*_1_ is still the PO strategy. (2) *k*_1_(*q*_*L*_) can be regarded as the lower limit of the R&D cost that *M*_1_ can bear. The larger the R&D cost is, the stronger the endurance will be. The negative correlation between *k*_1_(*q*_*L*_) and *q*_*L*_ means that a higher differentiation degree of CM product quality will lead to a weakened tolerance of *M*_1_ to the R&D cost. As mentioned in (1), the weak ability of upstream OEM manufacturers to produce differentiated products will inhibit the impulse of downstream enterprises to develop core technologies. Therefore, unless the cost is low, it is difficult to motivate *M*_1_ to invest in R&D, which is manifested by a reduced tolerance to R&D costs.

### 5.2 *M*_2_ procurement strategy selection

**Proposition 6**: Regardless of *M*_1_’s choice, the optimal procurement choice of *M*_2_ is as follows: when $c<\frac{1}{1+q_{L}}$, the HQ product is optimal ($\pi_{2}^{POHQ}>\pi_{2}^{POLQ}$, $\pi_{2}^{COHQ}>\pi_{2}^{COLQ}$); otherwise, when $c>\frac{1}{1+q_{L}}$, the LQ product is optimal ($\pi_{2}^{POHQ}<\pi_{2}^{POLQ}$, $\pi_{2}^{COHQ}<\pi_{2}^{COLQ}$).

**Fig 4:**
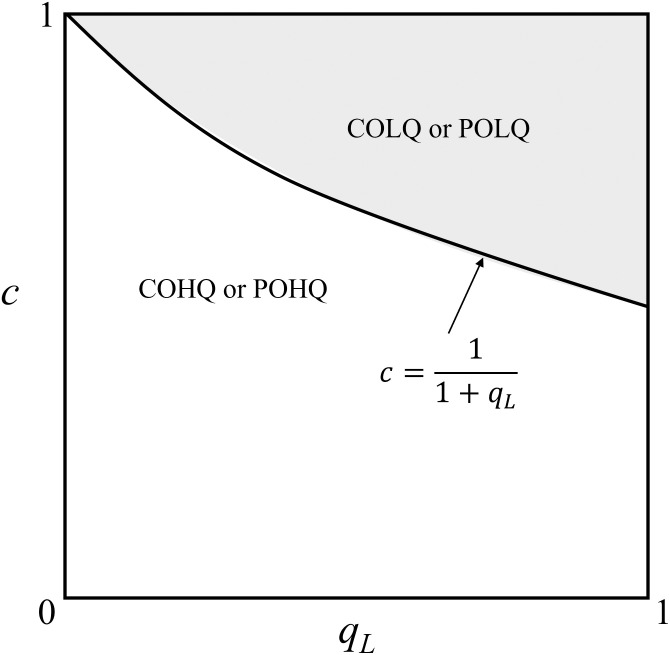
The influence of *c* and *q*_*L*_ on the maximal profit scenario of *M*_2_ (*a* = 1; *α* = 0.035; *m* = 0.4).

Proposition 6 presents how the quality cost *c* influences the optimal purchasing decision of *M*_2_ when *M*_1_ chooses the CO or PO strategy. For ease of understanding, we provide visualization in Fig 4 to illustrate the influence of *c* and *q*_*L*_ on the maximal profit scenario of *M*_2_. Note that Proposition 6 can be proofed mathematically (refer to Appendix 3 in S1 Appendix); hence, its parameter values do not impact Proposition 6. Also, the region of “COLQ or POLQ” in Fig 3 represents $\pi_{1}^{POLQ}>Max[\pi_{1}^{COLQ},\pi_{1}^{COHQ},\pi_{1}^{POHQ}]$. The same is true in case with “COHQ or POHQ”.

It is easy to find that a larger *c* means a higher wholesale cost of *M*_2_. Therefore, when *c* is small, the cost of purchasing high-quality products is relatively low. According to Proposition 3, when $c<\frac{1}{1+q_{L}}$, sales of high-quality products can obtain higher demand, so choosing the HQ strategy is more profitable. In contrast, when *c* is large ($c>\frac{1}{1+q_{L}}$), the purchase cost of high-quality products will increase, and the demand for high-quality products will be lower than that of low-quality products (see Proposition 3). Therefore, low-quality products should be purchased at this time, and the LQ strategy should be chosen.

**Proposition 7**: Compared with the PO mode, *M*_1_ choosing the CO mode will result in the loss of *M*_2_ revenue: $\pi_{2}^{COHQ}<\pi_{2}^{POHQ}$, $\pi_{2}^{COLQ}<\pi_{2}^{POLQ}$.

Proposition 7 illustrates the economic impact of *M*_1_’s choice of OEM mode on *M*_2_; that is, *M*_1_’s dominant product design will lead to a decline in *M*_2_’s revenue. In combination with proposition 2, *M*_1_ can obtain higher demand for products produced in the CO mode, but the market capacity is limited. A larger market share of *M*_1_ means a lower demand for *M*_2_ products, and the income of *M*_2_ will also be affected. Huawei, for example, has steadily increased its market share since it started selling phones with Kirin processors, becoming the top Chinese vendor of phone shipments in 2019, while sales of its main rival Xiaomi’s phones have been declining for years. The cases in the mobile phone market have proven that the enterprise mastering the core technology is bound to seize the market share of competitors, which will bring much pressure to their business. The reason is that, as shown in Proposition 2, the CO mode gives enterprises the dominant right in product design. Under the endogenous quality situation, enterprises can decide the optimal quality level according to the preferences of consumers and take the largest market share, which is obviously very harmful to competitors.

### 5.3 Influence of *M*_1_ and *M*_2_ strategies on the CM

**Proposition 8**: Regardless of the strategy choice of *M*_1_, the maximum profit scenario of the CM is consistent with that of *M*_2_: when $c<\frac{1}{1+q_{L}}$, $\pi_{CM}^{POHQ}>\pi_{CM}^{POLQ}$, $\pi_{CM}^{COHQ}>\pi_{CM}^{COLQ}$; when $c>\frac{1}{1+q_{L}}$, $\pi_{CM}^{POHQ}<\pi_{CM}^{POLQ}$, $\pi_{CM}^{COHQ}<\pi_{CM}^{COLQ}$.

**Fig 5:**
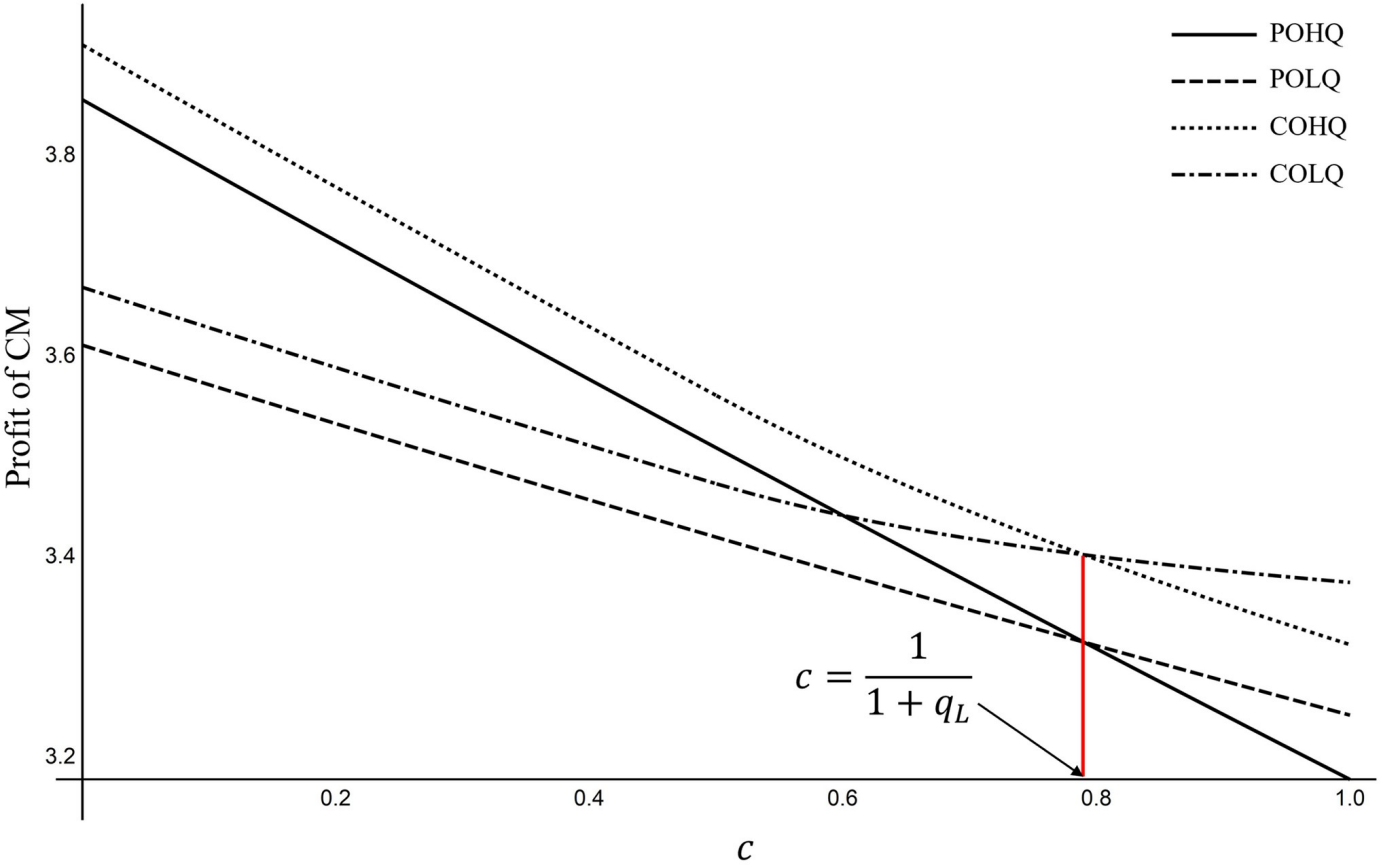
The influence of *c* on the profit of CM (*a* = 1; *α* = 0.9; *m* = 0.152; *q*_*L*_ = 0.266).

Proposition 8 shows the impact of *M*_2_’s purchasing strategy choice on CM revenue. Both have the same threshold value *c*. For ease of understanding, we provide a visualization in Fig 5 to show the influence of *c* on the profit of CM. Note that Proposition 8 can be proofed mathematically (refer to Appendix 3 in S1 Appendix); hence, its parameter values do not impact Proposition 8. When $c<\frac{1}{1+q_{L}}$, the production cost of high-quality products is not high, and it is easier to attract *M*_2_ to purchase. Moreover, the market demand of high-quality products is higher than that of low-quality products, and the CM will benefit from *M*_2_’s choice of HQ strategy. When $c>\frac{1}{1+q_{L}}$, the wholesale price of high-quality products will also rise, and *M*_2_ cannot afford high-price products but chooses low-quality products. Although the unit profit of low-quality products is far less than that of high-quality products, the sales volume is high. Meanwhile, the market demand of low-quality products is higher than that of high-quality products. CMs will also benefit from *M*_2_’s choice of LQ strategy.

**Proposition 9**: Regardless of the strategy choice of *M*_2_, CM can benefit from *M*_1_ choosing the CO mode: $\pi_{CM}^{COHQ}>\pi_{CM}^{POHQ}$, $\pi_{CM}^{COLQ}>\pi_{CM}^{POLQ}$.

**Fig 6:**
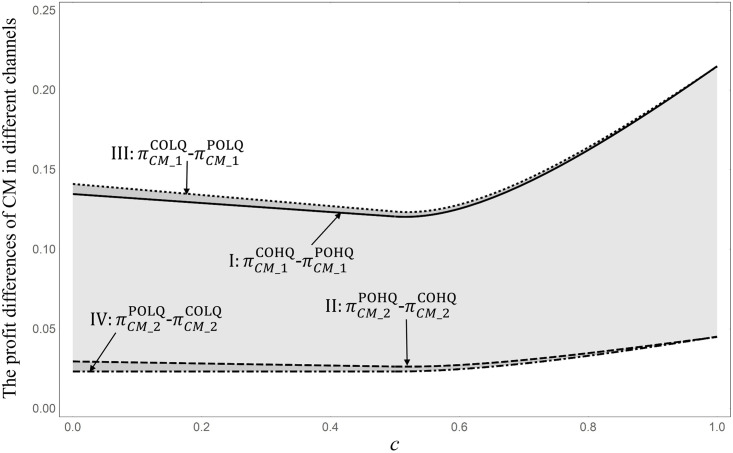
Impact of the *M*_1_ strategy choice on profit of the CM in different channels (*a* = 1; *α* = 0.742; *m* = 0.266; *q*_*L*_ = 0.017).

Proposition 9 points out the impact of *M*_1_’s choice of OEM mode on CM revenue; that is, regardless of what quality products *M*_2_ purchase, M1’s choice of OEM mode leading product design will always cause CMs to profit. However, Proposition 7 shows that *M*_1_’s choice of CO mode will lead to the loss of *M*_2_’s revenue, mainly because, in CO mode, *M*_1_ can determine the product quality that maximizes its profit, which will seriously erode *M*_2_’s market share. From this point of view, there is no doubt that the CO mode will reduce the CM’s income from *M*_2_, contrary to Proposition 9. For ease of understanding, Fig 6 help explain Proposition 9. Note that Proposition 9 can be proofed mathematically (see Appendix 8 in S1 Appendix). Therefore, its parameter values do not impact Proposition 9. Fig 6 illustrates the changes in profit of the CM’s two channels under different scenarios. $\pi_{CM\_1}^{COHQ}$ and $\pi_{CM\_2}^{COHQ}$ ($\pi_{CM\_1}^{COHQ}+\pi_{CM\_2}^{COHQ}=\pi_{CM}^{COHQ}$) represent the profits of CMs from the *M*_1_ and *M*_2_ channels, respectively, in the case of COLQ, and other symbols are used in the same way. Thus, we can identify the impact of the *M*_1_ strategy choice on CM’s revenue from different channels: I and II represent the increased profit of the *M*_1_ channel caused by the CO mode and the decreased profit of the *M*_2_ channel when *M*_2_ chooses the HQ strategy, respectively; III and IV represent the increased profits of the *M*_1_ channel caused by the CO mode and the decreased profits of the *M*_2_ channel when *M*_2_ chooses the LQ strategy, respectively. As shown in Fig 6, regardless of what quality products *M*_2_ purchase, the increased profit of the *M*_1_ channel is far greater than the decreased profit of the *M*_2_ channel. This explains why the CO mode hurts CM’s profitability in the *M*_2_ channel but still benefits it overall. The management implication of Proposition 9 reveals that if the contract manufacturer cannot directly obtain the relevant preference information from the end consumer, the optimal strategy is to provide only the CO mode. Without knowing consumer preferences, it is difficult to develop the products in line with market expectations, and the optimal decision of endogenous quality cannot be realized. At this point, downstream OEMs will lead to the development of core technologies. For contract manufacturers, it is most advantageous to conserve research and development investment funds and have higher market demand.

## 6. Conclusion

This paper conducts an in-depth study on the strategic interaction of original equipment manufacturers between their outsourcing and purchasing in a quality-differentiated market. Based on the decision order (game sequence) and the linear demand function assumed in section 4, we derive the optimal decisions and profits of two original equipment manufacturers and contract manufacturer. Finally, we analyse the influence of the OEM mode and quality differentiation on competitors and the whole supply chain by establishing and solving four decision models composed of two OEM modes (CO and PO) and two purchasing strategies with different quality configurations (HQ and LQ). Note that this paper is based on a stylized model rather than a data-driven empirical study, and the results of the paper are derived by mathematical derivation (see S1 Appendix). However, as mentioned in Section 3, the stylized model has been widely used in management science studies, especially supply chain management [31–35]. Although it does not involve real data, researchers can also derive some managerial insights. In fact, not relying on the data could help avoid the risk of overfitting.

The main results and managerial insights are summarized as follows: First, products under the CO mode can obtain higher demand than those under the PO mode. This means that the CO mode erodes the market share of competitive products and causes losses to competitors (Proposition 2). Second, whether the powerful manufacturer (*M*_1_) chooses the CO mode depends on the required and fixed R&D expenditure *k* (Proposition 5). If the cost is sufficiently high, the CO mode will be abandoned. However, the lower cost does not necessarily result in the adopter choosing the CO mode. The main reason is that the weakly differentiated product production capacity of upstream manufacturers will inhibit the impulse of downstream enterprises to research and develop core technologies. Third, the optimal purchasing strategy of the manufacturer (*M*_2_) is not affected by the OEM mode of the competitor but only depends on the production technology *c* of CM (Proposition 6): if it is too high, low-quality products will be chosen; otherwise, high-quality products are optimal. Fourth, regardless of *M*_1_’s OEM mode selection, the maximum profit scenario of the contract manufacturer is consistent with that of the disadvantaged manufacturer, which is also determined by the quality cost (Proposition 8). Fifth, for the contract manufacturer, there will be more advantages than disadvantages if the powerful manufacturer can dominate the decision on product quality (Proposition 9). This is because the downstream manufacturers directly contact consumers and have their preference information, which enables them to maximize their profits via the CO mode, although the contract manufacturer part channel gains will lead to damage to the increase of profit is much greater than the loss of profits.

By taking into account factors such as product quality differentiation, R&D investment and quality cost were taken into consideration, which contributes to enriching the prior studies regarding outsourcing and procurement strategies in the supply chain. There are several potential limitations in this paper. First, the influence of product quality uncertainty, production capacity and quality control fluctuation are not involved. Future research can consider the product quality uncertainty caused by PO mode OEM technology, production capacity or quality control fluctuation. Second, considering the impact of consumer preference changes on the choice of OEM mode under multiple cycles is worth further study. Finally, the stylized models of this paper are conducted based on industrial organization theory and game theory, and some empirical methods and real data can be introduced to further verify the conclusions in the future.

## Supporting information

S1 Appendix (DOCX)

S1 Code (TXT)
