# Supplementary material for: Strategic interaction of original equipment manufacturers between outsourcing and purchasing in a quality-differentiated market
Source: PLoS One. 2022 Jan 18;17(1):e0262678. doi: 10.1371/journal.pone.0262678 (PMC8765668; doi:10.1371/journal.pone.0262678)
Supplement: S1 Appendix — (DOCX) [file pone.0262678.s001.docx]

Appendix

**1. The optimal decisions and profits of all supply chain members**

**1.1 COHQ mode**

$q^{COHQ}=\left\{ \begin{aligned} 1,\frac{1}{2C}\geq1; \\ \frac{1}{2C},q_{L}<\frac{1}{2C}<1; \\ q_{L},\frac{1}{2C}\leq q_{L.} \end{aligned} \right.$ $w_{1}^{COHQ}=\left\{ \begin{aligned} \frac{1}{2}\left( 1+c+\frac{a}{1-\alpha} \right),\frac{1}{2c}\geq1; \\ \frac{3}{8c}+\frac{a}{2-2\alpha},q_{L}<\frac{1}{2c}<1; \\ \frac{1}{2}\left( q_{L}+cq_{L}^{2}+\frac{a}{1-\alpha} \right),\frac{1}{2c}\leq q_{L}. \end{aligned} \right.$ $w_{2}^{COHQ}=\frac{1}{2}\left( 1+c+m+\frac{a}{1-\alpha} \right)$

$$Q_{1}^{COHQ}\boldsymbol{=}\left\{ \begin{aligned} \frac{\left( 1+\alpha\right)\left[ a\left( \alpha-2 \right)+(\alpha-1)(2-2c+\alpha(c+m-1) \right]}{2(\alpha^{2}-4)},\frac{1}{2c}\geq1; \\ \frac{\left( 1+\alpha\right)\left[ 2ac\left( \alpha-2 \right)+(\alpha-1)(1+2c(c+m-1) \right]}{4c(\alpha^{2}-4)},q_{L}<\frac{1}{2c}<1; \\ \frac{\left( 1+\alpha\right)\left[ a\left( \alpha-2 \right)+(\alpha-1)(2q_{L}-2cq_{L}^{2}+\alpha(c+m-1) \right]}{2(\alpha^{2}-4)},\frac{1}{2c}\leq q_{L}. \end{aligned} \right.$$

$$Q_{2}^{COHQ}=\left\{ \begin{aligned} \frac{\left( 1+\alpha\right)\left[ a\left( \alpha-2 \right)+(\alpha-1)(\alpha c-\alpha-2(c+m-1) \right]}{2(\alpha^{2}-4)},\frac{1}{2c}\geq1; \\ \frac{\left( 1+\alpha\right)\left[ c\left( 4a\left( \alpha-2 \right)-8\left( 1-m \right)\left( 1-a \right) \right)+8c^{2}(1-\alpha)\alpha\right]}{8c(\alpha^{2}-4)},q_{L}<\frac{1}{2c}<1 \\ \frac{\left( 1+\alpha\right)\left[ a\left( \alpha-2 \right)+(\alpha-1)(c\alpha q_{L}^{2}-\alpha q_{L}-2(c+m-1) \right]}{2(\alpha^{2}-4)},\frac{1}{2c}\leq q_{L}. \end{aligned} \right.$$

$$\pi_{1}^{\mathrm{COHQ}}=\left\{ \begin{matrix} -k-\frac{(1+\alpha){(a(-2+\alpha)+(-1+\alpha)(2-2c+(-1+c+m)\alpha))}^{2}}{4(-1+\alpha){(-4+\alpha^{2})}^{2}} & \frac{1}{2c}\geq1 \\ \frac{\left[ \begin{aligned} 4a^{2}c^{2}\left( -2+\alpha\right)^{2}\left( 1+\alpha\right)+4ac\left( -2+\alpha\right)\left( -1+\alpha\right)\left( 1+\alpha\right)\left( 1+2c\left( -1+c+m \right)\alpha\right) \\ +(-1+\alpha)(-1+\alpha^{2}+4c(-1+m)\alpha(-1+\alpha^{2})+4c^{4}\alpha^{2}(-1+\alpha^{2})+ \\ 8c^{3}(-1+m)\alpha^{2}(-1+\alpha^{2})+4c^{2}(4k{(-4+\alpha^{2})}^{2}+\alpha(1+{(-1+m)}^{2}\alpha)(-1+\alpha^{2}))) \end{aligned} \right]}{-16c^{2}(-1+\alpha){(-4+\alpha^{2})}^{2}} & q_{L}\leq\frac{1}{2c}\leq1 \\ -k-\frac{(1+\alpha){(a(-2+\alpha)+(-1+\alpha)(-2q_{L}(-1+c\mathrm{ql})+(-1+c+m)\alpha))}^{2}}{4(-1+\alpha){(-4+\alpha^{2})}^{2}} & \frac{1}{2c}\leq q_{L} \end{matrix} \right.$$

$$\pi_{2}^{\mathrm{COHQ}}=\left\{ \begin{matrix} -\frac{(1+\alpha){(a(-2+\alpha)+(-1+\alpha)(-2(-1+c+m)+(-1+c)\alpha))}^{2}}{4(-1+\alpha){(-4+\alpha^{2})}^{2}} & \frac{1}{2c}\geq1 \\ -\frac{(1+\alpha){(c(-4a(-2+\alpha)+8(-1+m)(-1+\alpha))+8c^{2}(-1+\alpha)+(-1+\alpha)\alpha)}^{2}}{64c^{2}(-1+\alpha){(-4+\alpha^{2})}^{2}} & q_{L}\leq\frac{1}{2c}\leq1 \\ -\frac{(1+\alpha){(a(-2+\alpha)+(-1+\alpha)(-2(-1+c+m)+q_{L}(-1+cq_{L})\alpha))}^{2}}{4(-1+\alpha){(-4+\alpha^{2})}^{2}} & \frac{1}{2c}\leq q_{L} \end{matrix} \right.$$

$$\pi_{\mathrm{CM}}^{\mathrm{COHQ}}=\left\{ \begin{matrix} \frac{(1+\alpha)\left[ \begin{aligned} a^{2}(-2+\alpha)+a(-2+2c+m)(-2+\alpha)(-1+\alpha)+\left( -1+\alpha\right)^{2}(-2+ \\ c^{2}(-2+\alpha)+c(-2+m)(-2+\alpha)+\alpha-m(-2+m+\alpha)) \end{aligned} \right]}{2(1-\alpha)(-4+\alpha^{2})} & \frac{1}{2c}\geq1 \\ \frac{(1+\alpha)\left[ \begin{aligned} -16a^{2}c^{2}\left( -2+\alpha\right)-4ac\left( -1+4c\left( -1+c+m \right) \right)\left( -2+\alpha\right)\left( -1+\alpha\right)+ \\ {(-1+\alpha)}^{2}(1+4c(-1+c+m)(4c(-1+c+m)+\alpha)) \end{aligned} \right]}{32c^{2}(-1+\alpha)(-4+\alpha^{2})} & q_{L}\leq\frac{1}{2c}\leq1 \\ \frac{(1+\alpha)\left[ \begin{aligned} \left( a+q_{L}\left( -1+c\mathrm{ql} \right)\left( -1+\alpha\right) \right) \\ \left( a\left( -2+\alpha\right)+\left( -1+\alpha\right)\left( 2q_{L}-2c{q_{L}}^{2}+\left( -1+c+m \right)\alpha\right) \right)+ \\ (a+(-1+c+m)(-1+\alpha))(a(-2+\alpha)+ \\ (-1+\alpha)(-2(-1+c+m)+q_{L}(-1+cq_{L})\alpha)) \end{aligned} \right]}{4(1-\alpha)(-4+\alpha^{2})} & \frac{1}{2c}\leq q_{L} \end{matrix} \right.$$

**1.2 COLQ mode**

$q^{COLQ}=\left\{ \begin{aligned} 1,\frac{1}{2C}\geq1; \\ \frac{1}{2C},q_{L}<\frac{1}{2C}<1; \\ q_{L},\frac{1}{2C}\leq q_{L.} \end{aligned} \right.$ $w_{1}^{COLQ}=\left\{ \begin{aligned} \frac{1}{2}\left( 1+c+\frac{a}{1-\alpha} \right),\frac{1}{2c}\geq1; \\ \frac{3}{8c}+\frac{a}{2-2\alpha},q_{L}<\frac{1}{2c}<1; \\ \frac{1}{2}\left( q_{L}+cq_{L}^{2}+\frac{a}{1-\alpha} \right),\frac{1}{2c}\leq q_{L}. \end{aligned} \right.$ $w_{2}^{COLQ}=\frac{1}{2}\left( m+q_{L}+cq_{L}^{2}+\frac{a}{1-\alpha} \right)$

$$Q_{1}^{COLQ}\boldsymbol{=}\left\{ \begin{aligned} \frac{\left( 1+\alpha\right)\left[ a\left( \alpha-2 \right)+(\alpha-1)(2-2c+(m+q_{L}\left( cq_{L}-1 \right))\alpha\right]}{2(\alpha^{2}-4)},\frac{1}{2c}\geq1; \\ \frac{\left( 1+\alpha\right)\left[ 2ac\left( \alpha-2 \right)+(\alpha-1)(1+2c(m+q_{L}\left( cq_{L}-1 \right))\alpha\right]}{4c(\alpha^{2}-4)},q_{L}<\frac{1}{2c}<1; \\ \frac{\left( 1+\alpha\right)\left[ a\left( \alpha-2 \right)+(\alpha-1)(q_{L}(cq_{L}-1)(\alpha-2)+m\alpha\right]}{2(\alpha^{2}-4)},\frac{1}{2c}\leq q_{L}. \end{aligned} \right.$$

$$Q_{2}^{COlQ}=\left\{ \begin{aligned} \frac{\left( 1+\alpha\right)\left[ a\left( \alpha-2 \right)+(\alpha-1)(\alpha c-\alpha-2(m+q_{L}\left( cq_{L}-1 \right)) \right]}{2(\alpha^{2}-4)},\frac{1}{2c}\geq1; \\ \frac{\left( 1+\alpha\right)\left[ \left( 4ac\left( \alpha-2 \right)-(\alpha-1)(8c\left( m+q_{L}\left( cq_{L}-1 \right) \right)+\alpha\right) \right]}{8c(\alpha^{2}-4)},q_{L}<\frac{1}{2c}<1 \\ \frac{\left( 1+\alpha\right)\left[ a\left( \alpha-2 \right)+(\alpha-1)((cq_{L}^{2}-q_{L})(\alpha-2)-2m \right]}{2(\alpha^{2}-4)},\frac{1}{2c}\leq q_{L}. \end{aligned} \right.$$

$$\pi_{1}^{\mathrm{COLQ}}=\left\{ \begin{matrix} -k-\frac{(1+\alpha){(a(-2+\alpha)+(-1+\alpha)(2-2c+m\alpha-q_{L}\alpha+c{q_{L}}^{2}\alpha))}^{2}}{4(-1+\alpha){(-4+\alpha^{2})}^{2}} & \frac{1}{2c}\geq1 \\ -k-\frac{(1+\alpha){(2ac(-2+\alpha)+(-1+\alpha)(1+2c(m+q_{L}(-1+cq_{L}))\alpha))}^{2}}{16c^{2}(-1+\alpha){(-4+\alpha^{2})}^{2}} & q_{L}\leq\frac{1}{2c}\leq1 \\ -k-\frac{(1+\alpha){(a(-2+\alpha)+(-1+\alpha)(q_{L}(-1+cq_{L})(-2+\alpha)+m\alpha))}^{2}}{4(-1+\alpha){(-4+\alpha^{2})}^{2}} & \frac{1}{2c}\leq q_{L} \end{matrix} \right.$$

$$\pi_{2}^{\mathrm{COLQ}}=\left\{ \begin{matrix} -\frac{(1+\alpha){(a(-2+\alpha)+(-1+\alpha)(-2(m+q_{L}(-1+cq_{L}))+(-1+c)\alpha))}^{2}}{4(-1+\alpha){(-4+\alpha^{2})}^{2}} & \frac{1}{2c}\geq1 \\ -\frac{(1+\alpha){(-4ac(-2+\alpha)+(-1+\alpha)(8c(m+q_{L}(-1+cq_{L}))+\alpha))}^{2}}{64c^{2}(-1+\alpha){(-4+\alpha^{2})}^{2}} & q_{L}\leq\frac{1}{2c}\leq1 \\ -\frac{{(a(-2+\alpha)+(-2m+q_{L}(-1+cq_{L})(-2+\alpha))(-1+\alpha))}^{2}(1+\alpha)}{4(-1+\alpha){(-4+\alpha^{2})}^{2}} & \frac{1}{2c}\leq q_{L} \end{matrix} \right.$$

$$\pi_{\mathrm{CM}}^{\mathrm{COLQ}}=\left\{ \begin{matrix} \frac{(1+\alpha)\left[ \begin{aligned} 2a^{2}\left( -2+\alpha\right)+2a\left( -1+c+m-q_{L}+c{q_{L}}^{2} \right)\left( -2+\alpha\right)\left( -1+\alpha\right)-2\left( -1+\alpha\right)^{2} \\ ({(-1+c)}^{2}+(m+q_{L}(-1+cq_{L}))(m+q_{L}(-1+cq_{L})+\alpha-c\alpha)) \end{aligned} \right]}{4(1-\alpha)(-4+\alpha^{2})} & \frac{1}{2c}\geq1 \\ \frac{(1+\alpha)\left[ \begin{aligned} -16a^{2}c^{2}\left( -2+\alpha\right)-4ac\left( -1+4c\left( m+\mathrm{ql}\left( -1+cq_{L} \right) \right) \right)\left( -2+\alpha\right)\left( -1+\alpha\right)+ \\ {(-1+\alpha)}^{2}(1+4c(m+q_{L}(-1+cq_{L}))(4c(m+q_{L}(-1+cq_{L}))+\alpha)) \end{aligned} \right]}{32c^{2}(-1+\alpha)(-4+\alpha^{2})} & q_{L}\leq\frac{1}{2c}\leq1 \\ \frac{(1+\alpha)\left[ \begin{aligned} a^{2}(-2+\alpha)+a(m+2q_{L}(-1+cq_{L}))(-2+\alpha)(-1+\alpha)+(-m^{2}+ \\ mq_{L}(-1+cq_{L})(-2+\alpha)+{q_{L}}^{2}{(-1+cq_{L})}^{2}(-2+\alpha)){(-1+\alpha)}^{2} \end{aligned} \right]}{2(1-\alpha)(-4+\alpha^{2})} & \frac{1}{2c}\leq q_{L} \end{matrix} \right.$$

**1.3 POHQ mode**

$w_{1}^{POHQ}=w_{2}^{POHQ}=\frac{1}{2}\left( 1+c+m+\frac{a}{1-\alpha} \right);$ $Q_{1}^{POHQ}=Q_{1}^{POHQ}=\frac{(1+\alpha)\left[ a+(c+m-1)(+\alpha-1) \right]}{2(2+\alpha)}$;

$\pi_{1}^{POHQ}=\pi_{2}^{POHQ}=\frac{\left[ a+\left( c+m-1 \right)\left( \alpha-1 \right) \right]^{2}(1+\alpha)}{4(1-\alpha){(2+\alpha)}^{2}}$, $\pi_{CM}^{POHQ}=\frac{\left[ a+\left( c+m-1 \right)\left( \alpha-1 \right) \right]^{2}(1+\alpha)}{2(2-\alpha-\alpha^{2})}$.

**1.4 POLQ mode**

$w_{1}^{POLQ}=\frac{1}{2}\left( 1+c+m+\frac{a}{1-\alpha} \right)$, $w_{2}^{POLQ}=\frac{1}{2}\left( m+q_{L}+cq_{L}^{2}+\frac{a}{1-\alpha} \right)$;

$Q_{1}^{POLQ}=\frac{(1+\alpha)\left[ a\left( \alpha-2 \right)+(\alpha-1)\left( \left( m+q_{L}\left( cq_{L}-1 \right) \right)\alpha-2(c+m-1) \right) \right]}{{2(\alpha}^{2}-4)}$;

$Q_{2}^{POLQ}=\frac{(1+\alpha)\left[ a\left( \alpha-2 \right)+(\alpha-1)\left( \left( c+m-1 \right)\alpha-2\left( m+q_{L}\left( cq_{L}-1 \right) \right) \right) \right]}{{2(\alpha}^{2}-4)}$;

$\pi_{1}^{POLQ}=\frac{(1+\alpha)\left[ a\left( \alpha-2 \right)+(\alpha-1)\left( \left( m+q_{L}\left( cq_{L}-1 \right) \right)\alpha-2(c+m-1) \right) \right]^{2}}{4\left( 1-\alpha\right)\left( \alpha^{2}-4 \right)^{2}}$;

$\pi_{2}^{POLQ}=\frac{(1+\alpha)\left[ a\left( \alpha-2 \right)+(\alpha-1)\left( \left( c+m-1 \right)\alpha-2\left( m+q_{L}\left( cq_{L}-1 \right) \right) \right) \right]^{2}}{4\left( 1-\alpha\right)\left( \alpha^{2}-4 \right)^{2}}$;

$\pi_{CM}^{POLQ}=\left[ \left( a+\left( m+q_{L}\left( cq_{L}-1 \right) \right)\left( \alpha-1 \right) \right)\left( \alpha\left( \alpha-2 \right)+\left( \alpha-1 \right)\left( \left( c+m-1 \right)\alpha-2\left( m+q_{L}\left( cq_{L}-1 \right) \right) \right) \right)+\left( a+\left( c+m-1 \right)\left( \alpha-1 \right) \right)\left( a\left( \alpha-2 \right)+\left( \alpha-1 \right)\left( \left( m+q_{L}\left( cq_{L}-1 \right) \right)\alpha-2\left( c+m-1 \right) \right) \right) \right]/4\left( 1-\alpha\right)\left( \alpha^{2}-4 \right)^{2}$.

**2. Proof of Proposition 2**

If $q_{L}<\frac{1}{2C}<1$, then $Q_{1}^{COLQ}-Q_{1}^{POLQ}=\frac{\left( 1+4c\left( c+m-1 \right)+2c\left( 1-q_{L} \right)\left( c+cq_{L}-1 \right)\alpha\right)\left( 1-\alpha^{2} \right)}{4c\left( 4-\alpha^{2} \right)}>0$ and $Q_{1}^{COLQ}-Q_{1}^{POLQ}=\frac{\left( 1+4c\left( c+m-1 \right) \right)\left( 1-\alpha^{2} \right)}{4c\left( 4-\alpha^{2} \right)}>0$; in the same way, the conclusion is still true for cases $\frac{1}{2c}\leq q_{L}$ and $\frac{1}{2c}\geq1$.

**3. Proof of Proposition 3**

To solve equation $Q_{2}^{POHQ}-Q_{2}^{POLQ}=0$, it has a unique real root $c=\frac{1}{1+q_{L}}$; since $\frac{\partial(Q_{2}^{POHQ}-Q_{2}^{POLQ})}{\partial c}=\frac{\left( q_{L}^{2}-1 \right)\left( 1-\alpha^{2} \right)}{4-\alpha^{2}}<0$, we have the following: when$c<\frac{1}{1+q_{L}}$, $Q_{2}^{POHQ}>Q_{2}^{POLQ}$; or when $c>\frac{1}{1+q_{L}}$, $Q_{2}^{POHQ}<Q_{2}^{POLQ}$. Similarly, we can compare $Q_{2}^{COHQ}$ and $Q_{2}^{COLQ}$.

**4. Proof of Proposition 4**

When $q_{L}<\frac{1}{2c}<1$, since $w_{1}^{POHQ}=w_{1}^{POLQ}$ and $w_{1}^{COHQ}=w_{1}^{COLQ}$, we have $w_{1}^{POHQ}-w_{1}^{COHQ}=w_{1}^{POLQ}-w_{1}^{COLQ}=\frac{4c\left( 1+c+m \right)-3}{8c}>0$; since $w_{2}^{COHQ}=w_{2}^{POHQ}$ and $w_{2}^{COLQ}=w_{2}^{POLQ}$, we have $w_{2}^{COHQ}-w_{2}^{COLQ}=w_{2}^{POHQ}-w_{2}^{POLQ}=\frac{1}{2}\left( 1-q_{L} \right)\left( 1+c+cq_{L} \right)>0$. In the same way, the conclusion is still true for cases $\frac{1}{2c}\leq q_{L}$ and $\frac{1}{2c}\geq1$.

**5. Proof of Proposition 5**

When $\frac{1}{2c}\leq q_{L}$, solving equation $\pi_{1}^{COLQ}-\pi_{1}^{POLQ}=0$ has a unique real root $k_{4}=\frac{\left( 1+\alpha\right)\left[ \left( a\left( -2+\alpha\right)+\left( -1+\alpha\right)\left( -2\left( -1+c+m \right)+\left( m+q_{L}\left( -1+cq_{L} \right) \right)\alpha\right) \right)^{2}-\left( a\left( -2+\alpha\right)+\left( -1+\alpha\right)\left( q_{L}\left( -1+cq_{L} \right)\left( -2+\alpha\right)+m\alpha\right) \right)^{2} \right]}{4\left( \alpha-1 \right)\left( \alpha^{2}-4 \right)^{2}}$; since $\frac{\partial(\pi_{1}^{COHQ}-\pi_{1}^{POHQ})}{\partial k}=-1<0$, we have the following: when $k<k_{4}$,$\pi_{1}^{COLQ}>\pi_{1}^{POLQ}$; or when $k>k_{4}$, $\pi_{1}^{COLQ}<\pi_{1}^{POLQ}$. In the same way, similar conclusions can be proven (the relevant thresholds are as follows).

$k_{1}=\frac{-\left( -1+c+m+q_{L}-c{q_{L}}^{2} \right)\left( 1+\alpha\right)\left( a\left( -2+\alpha\right)-\left( -1+\alpha\right)\left( -1+m-q_{L}+c\left( 1+{q_{L}}^{2}-\alpha\right)+\alpha-m\alpha\right) \right)}{\left( \alpha^{2}-4 \right)^{2}}$;

$k_{2}=\frac{-\left( 1+4c\left( -1+c+m \right) \right)\left( 1+\alpha\right)\left( -1+4c\left( a\left( -2+\alpha\right)+\left( -1+c+m \right)\left( -1+\alpha\right)^{2} \right)+\alpha\right)}{16c^{2}\left( \alpha^{2}-4 \right)^{2}}$;

$k_{3}=\frac{-m\left( 1+\alpha\right)\left( a\left( -2+\alpha\right)+\left( -1+\alpha\right)\left( 2-2c-m+\left( -1+c+m \right)\alpha\right) \right)}{\left( \alpha^{2}-4 \right)^{2}}$;

$k_{5}=\frac{-\left( 1+4c\left( -1+c+m \right) \right)\left( 1+\alpha\right)\left( -1+\alpha+4c\left( a\left( -2+\alpha\right)+\left( -1+\alpha\right)\left( 1-c-m+\left( m+q_{L}\left( -1+cq_{L} \right) \right)\alpha\right) \right) \right)}{16c^{2}\left( \alpha^{2}-4 \right)^{2}}$;

$k_{6}=\frac{-m\left( 1+\alpha\right)\left( a\left( -2+\alpha\right)+\left( -1+\alpha\right)\left( 2-2c-m+\left( m+q_{L}\left( -1+cq_{L} \right) \right)\alpha\right) \right)}{\left( \alpha^{2}-4 \right)^{2}}$.

**6. Proof of Proposition 6 and 8**

Similar to Proposition 3, it is easy to prove propositions 6 and 8.

**7. Proof of Proposition 7**

When$\frac{1}{2c}\geq1$, $\pi_{2}^{COHQ}-\pi_{2}^{POHQ}=\frac{m\alpha\left( 1+\alpha\right)\left( 2a\left( \alpha-2 \right)+\left( \alpha-1 \right)\left( -4\left( c+m-1 \right)+\left( 2c+m-2 \right)\alpha\right) \right)}{4\left( \alpha^{2}-4 \right)^{2}}<0$; then,$\pi_{2}^{COHQ}<\pi_{2}^{POHQ}$. In the same way, the conclusion is still true for cases $\frac{1}{2c}\leq q_{L}$ and $q_{L}<\frac{1}{2C}<1$.

**8. Proof of Proposition 9**

(1) When$\frac{1}{2c}\geq1$, $\pi_{CM}^{POHQ}-\pi_{CM}^{COHQ}=-\frac{m\left( 1+\alpha\right)\left( a\left( -2+\alpha\right)+\left( -1+\alpha\right)\left( 2-2c-m+\left( -1+c+m \right)\alpha\right) \right)}{2\left( -4+\alpha^{2} \right)}<0$; then,$\pi_{CM}^{COHQ}>\pi_{CM}^{POHQ}$. In the same way, the conclusion is still true for cases $\frac{1}{2c}\leq q_{L}$ and $q_{L}<\frac{1}{2C}<1$.

(2) When$\frac{1}{2c}\geq1$, $\pi_{CM}^{POLQ}-\pi_{CM}^{COLQ}=-\frac{m(1+\alpha)(a(-2+\alpha)+(-1+\alpha)(2-2c-m+(m+q_{L}(-1+cq_{L}))\alpha))}{2(-4+\alpha^{2})}<0$; then,$\pi_{CM}^{COLQ}>\pi_{CM}^{POLQ}$. In the same way, the conclusion is still true for cases $\frac{1}{2c}\leq q_{L}$ and $q_{L}<\frac{1}{2C}<1$.

**9. Proof of jointly concave function of CM’s profit with respect to** $\boldsymbol{w}_{\boldsymbol{1}}$ **and** $\boldsymbol{w}_{\boldsymbol{2}}$

To verify that the optimal solutions of $w_{1}$ and $w_{2}$ exist simultaneously, taking the partial derivative of $\pi_{CM}=\left( w_{1}-cq^{2} \right)Q_{1}+(w_{2}-c-m)Q_{1}$ with respect to $w_{1}$ and $w_{2}$, we can obtain the Hessian Matrix:

$$H_{\pi_{CM}(w_{1},w_{2})}^{(CO)}=\left[ \begin{matrix} -\frac{4(-1+\alpha^{2})}{-4+\alpha^{2}} & \frac{2\alpha(-1+\alpha^{2})}{-4+\alpha^{2}} \\ \frac{2\alpha(-1+\alpha^{2})}{-4+\alpha^{2}} & -\frac{4(-1+\alpha^{2})}{-4+\alpha^{2}} \end{matrix} \right]$$

where since $0<\alpha<1$, the first-order principal minor is $\frac{\partial\pi_{CM}^{2}}{\partial w_{1}^{2}}=-\frac{4(-1+\alpha^{2})}{-4+\alpha^{2}}<0$ and the second-order principal minor is $\frac{\partial\pi_{CM}^{2}}{\partial w_{1}^{2}}\cdot\frac{\partial\pi_{CM}^{2}}{\partial w_{2}^{2}}-\frac{\partial\pi_{CM}^{2}}{\partial w_{1}\partial w_{2}}\cdot\frac{\partial\pi_{CM}^{2}}{\partial w_{2}\partial w_{1}}=-\frac{4{(-1+\alpha^{2})}^{2}}{-4+\alpha^{2}}>0$, so $H_{\pi_{CM}(w_{1},w_{2})}^{(CO)}$ is a negative definite matrix. Therefore, this $\pi_{CM}(w_{1}, w_{2})$ is a jointly concave function with respect to $w_{1}$ and $w_{2}$.
